# Biological Characteristics of a Novel Bibenzyl Synthase (*DoBS1*) Gene from *Dendrobium officinale* Catalyzing Dihydroresveratrol Synthesis

**DOI:** 10.3390/molecules29225320

**Published:** 2024-11-12

**Authors:** Shao-Guo Zhou, Ke Zhong, Feng-Xia Yan, Fan Tian, Chang-Sha Luo, Hang-Cheng Yu, Zai-Qi Luo, Xi-Min Zhang

**Affiliations:** 1 College of Pharmacy, Guizhou University of Traditional Chinese Medicine, Guiyang 550025, China; zsg1721@126.com (S.-G.Z.); lionzhongke@163.com (K.Z.); 2 Key Laboratory for Biodiversity Conservation in Karst Mountain Area of Southwestern China, National Foresty and Grassland Administration, Guiyang 550005, China; yfx19871017@163.com (F.-X.Y.); tianfan850507@126.com (F.T.); lcs1314999@163.com (C.-S.L.); yhc942569@163.com (H.-C.Y.); 3 Guizhou Academy of Forestry, Nanming District, Guiyang 550005, China

**Keywords:** bibenzyl synthase, *Dendrobium officinale*, dihydroresveratrol, enzyme activity, in vitro biosynthesis

## Abstract

Bibenzyl compounds are one of the most important bioactive components of natural medicine. However, *Dendrobium officinale* as a traditional herbal medicine is rich in bibenzyl compounds and performs functions such as acting as an antioxidant, inhibiting cancer cell growth, and assisting in neuro-protection. The biosynthesis of bibenzyl products is regulated by bibenzyl synthase (BBS). In this study, we have cloned the cDNA gene of the bibenzyl synthase (*DoBS1*) from *D. officinale* using PCR with degenerate primers, and we have identified a novel type III polyketide synthase (PKS) gene by phylogenetic analyses. In a series of perfect experiments, DoBS1 was expressed in Escherichia coli, purified and some catalytic properties of the recombinant protein were investigated. The molecular weight of the recombinant protein was verified to be approximately 42.7 kDa. An enzyme activity analysis indicated that the recombinant DoBS1-HisTag protein was capable of using 4-coumaryol-CoA and 3 malonyl-CoA as substrates for dihydroresveratrol (DHR) in vitro. The Vmax and Km of the recombinant protein for DHR were 3.57 ± 0.23 nmol·min^−1^·mg^−1^ and 0.30 ± 0.08 mmol, respectively. The present study provides further insights into the catalytic mechanism of the active site in the biosynthetic pathway for the catalytic production of dihydroresveratrol by bibenzylase in *D. officinale*. The results can be used to optimize a novel biosynthetic pathway for the industrial synthesis of DHR.

## 1. Introduction

Bibenzyl compounds, a key component of the traditional Chinese medicinal herb, are naturally synthesized in *Dendrobium* plants. They consist of two phenylene groups linked by ethane (C6-C2-C6) [1,2]. This motif has attracted considerable interest due to their significant and diverse pharmacological activities. Several studies have shown that they include anti-tumor [3,4], anti-diabetic complications [5], neuroprotective [6,7], antioxidant [8], anti-inflammatory [9], antifungal [10], antiplatelet aggregation [11] and immunomodulatory activities [12,13]. The compounds have a wide range of biological activities and have simple structures, abundant substituents, and diverse skeletal linkages [14]. Between 1981 and 2019, the U.S. Food and Drug Administration approved a total of 1394 new small-molecule drugs for marketing. A significant majority, exceeding 60%, of small-molecule drugs have origins in natural products. A noteworthy example includes paclitaxel, and it is important to note that up to 90% of approved antifungal drugs are derived from natural sources [15]. This suggests promising prospects for the future development of bibenzyl compounds.

These bibenzyl compounds are scarce throughout the plant kingdom and are only known to accumulate in some members of the Orchidaceae family and in a few species of liverworts [1,16,17,18]. Only a handful of bibenzyl synthase have been reported and characterized as essential enzymes for the biosynthesis of bibenzyls, such as PsBBS from *Pinus sylvestris*, pBibsy811, and pBibsy212 from *Phalaenopsis* sp., and DsBBS from *Dendrobium sinense*, with the same selectivity toward m-hydroxyphenylpropionyl-CoA [19,20,21]. Given the high biological activity of bibenzyls, it is crucial to elucidate the pathways involved in their biosynthesis. The conservation of the Cys-His-Asn catalytic triad across all known type III polyketide synthases (PKSs) indicates that homologous genes may share similar functions [22]. In addition, differences in amino acid sequences are likely to account for the structural and functional diversity observed in type III PKSs.

**Figure 1:**
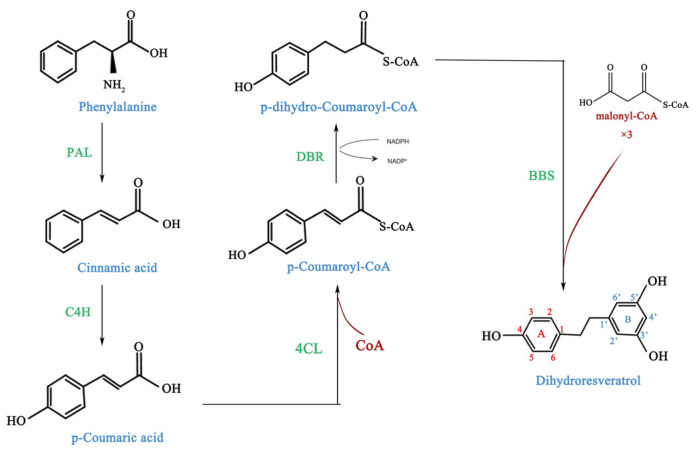
The proposed biosynthetic pathway for bibenzyls. PAL: phenylalanine ammonia-lyase; C4H: cinnamic-4-hydroxylase; 4CL: 4-coumaryl-CoA ligase; DBR: double-bond reductases; BBS: bibenzyl synthase.

Type III PKSs catalyze cyclization and aromatization of intermediate forms of polyketides [23]. Bibenzyls, which belong to the polyketide family, are derived from the phenylpropanoid biosynthetic pathway. This pathway is responsible for the biosynthesis of many secondary metabolites [24]. The different directions of phenylalanine metabolism have significant effects on the accumulation of secondary metabolites and the development of plant cells [25]. Bibenzyls are bicyclic intermediates. Previous radio-labeling studies in orchids have suggested a reaction sequence for bibenzyls synthesis that mirrors the mechanism used for the assembly of flavonoids and stilbenes in most plants by polyketide synthase. The ‘A-ring’ of the bibenzyl structure is derived from a hydroxycinnamic acid ‘starter molecule’ sourced from the phenylpropanoid pathway. Following esterification to coenzyme A (CoA), the starter molecule undergoes iterative condensation with three molecules of malonyl-CoA, resulting in the formation of a linear tetraketide intermediate. This is followed by intramolecular aldol cyclization, which completes the ‘B-ring’ of the bibenzyl scaffold (Figure 1) [26].

*Dendrobium officinale*, a member of the orchid family, is a type of traditional Chinese medicinal herb [27,28]. The species is widely distributed in Southeast China and Southeast Asian countries [29]. The pharmacological properties of the genus *Dendrobium* have been historically valued. Phytochemical analysis has revealed a wide range of bioactive compounds in *Dendrobium* plants [30]. Research focuses on exploring the pharmacological properties of bibenzyls on *Dendrobium* species. For example, 4,5-dihydroxy-3,3,4-trimethoxy bibenzyl isolated from *D. lindleyi* has an inhibitory effect on lung cancer growth and metastasis [12]. The bibenzyl components from *D. falconeri* may promote the expression of integrin, which inhibits epithelial–mesenchymal transition to block the migration and proliferation of lung cancer cells [31]. Bibenzyl compounds, as the major active constituents of *D. officinale*, have demonstrated pharmacological functions, such as antioxidant and anti-human pathogenic properties [27,32,33].

Due to their low abundance in nature, uncontrollable and environmentally unfriendly chemical synthesis processes, these compounds are not readily accessible, thus failing to meet market demand. Consequently, while chemical synthesis methods are being developed, they contradict the green principles of modern production. At the same time, biosynthesis has emerged as a favored approach for material acquisition [34]. Recent research has made new progress on structural diversification of bioactive bibenzyls through modular co-culture, leading to the discovery of a novel neuroprotective agent [34,35]. In addition, molecular docking revealed small differences in the amino acids of the enzyme protein leading to differences in catalytic efficiency of the catalytic roles of DsBBS1 and DsBBS2 in the bibenzylic biosynthesis of *Dendrobium sinense* [36]. There is an urgent need for a sustainable source of plant derived bibenzyl, and *D. officinale* has excellent potential for the production of these bibenzyl compounds. However, the physiological and molecular mechanisms underlying bibenzyl biosynthesis in *D. officinale* remains poorly understood. Therefore, it is desirable to reveal these mechanisms and gain a deeper understanding of the catalytic activity of amino acid sites to solve the rapid, efficient, and accurate biosynthesis of bibenzyl compounds, which will provide a basic framework for the industrial synthesis of dihydroresveratrol.

## 2. Results

### 2.1. Isolation of the DoBS1

A total of 12 gene fragments were amplified using the degenerate primers listed in Table S1, followed by a homologous comparison in the database. Among these amplified fragments, three specific gene fragments were found to be associated with the benzyl synthase gene, as detailed in Tables S2 and S10–S12. The homology alignment diagram for these three specific gene fragments is presented in the Supplementary Material (Figures S1–S3), revealing that only two of the fragments exhibited over 90% homology with the BBS sequence. Notably, the 12th gene fragment in Table S2 displayed the highest number of sequences, exceeding 90% homology to the BBS. Consequently, the 12th gene fragment was selected for full-length amplification in this study. PCR amplification was performed using *D. officinale* cDNA as a template with RTDoBS1-F and RTDoBS1-R primers. Subsequent analysis of the PCR product was performed through agarose gel electrophoresis, showing a distinct DNA band of 1000–1200 bp in size (Figure S4). Following this, the PCR product was recovered and sequenced, with the specific sequencing results presented in Figure S5. The gene was designated as *DoBS1*.

### 2.2. Sequence Analysis

The product sequence was compared to the expected sequence and analysis of the sequencing results using DNAMAN 6.0 software revealed it was called *DoBS1*. The sequencing data have been submitted in the NCBI database under accession number WDI23477. The open reading frame (ORF) spans 1182 bp and encodes 390 amino acids. The nucleotide sequence represents the coding region, while the amino acid sequence is shown in bold (Figure S6).

**Figure 2:**
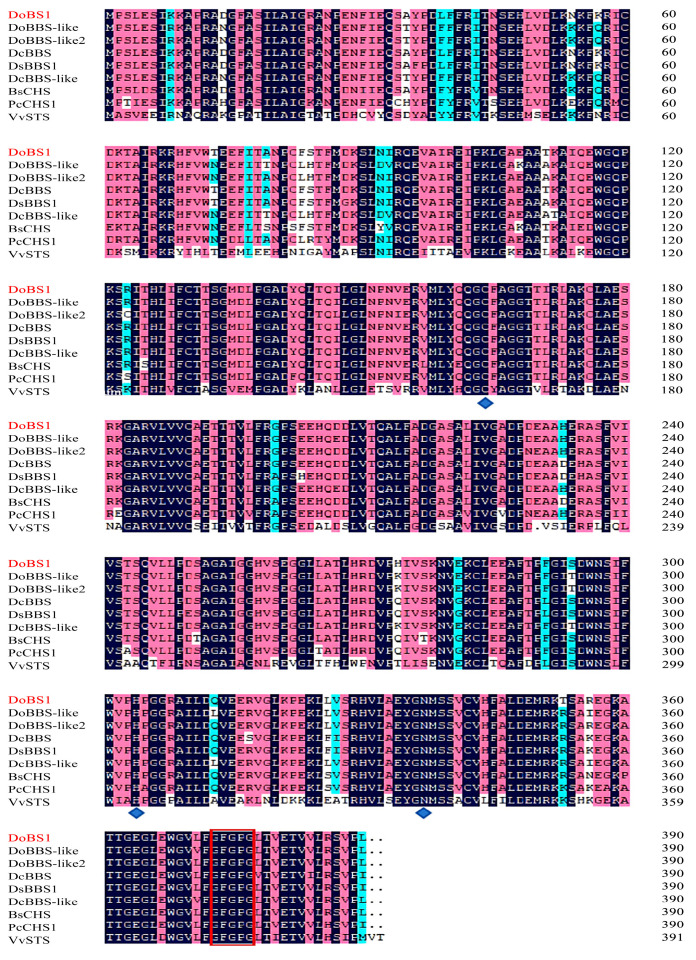
Amino acid sequence comparison of six type III PKSs of plant origin. A multiple sequence alignment was calculated with the DNAMAN package. Black shading indicates the homology of amino acids, while red and blue shading indicates amino acids with different similarity. The conserved catalytic residues in plant type III PKS (Cys164, His304 and Asn337, DoBS1 numbers) are represented by diamonds. Additionally, the highly conserved sequence G373FGPG (DoBS1 number) is indicated by red boxes.

Sequence alignment results indicated that the cloned DoBS1 amino acid sequence shared 94.36%, 95.38%, 96.67%, 95.64%, 94.36%, 91.54%, 89.23%, and 37.69% identity with the *DoBBS-like* (QCO76957.1), *DoBBS-like2* (WOK44117.1), *DcBBS* (XP 020704098.1), *DsBBS1* (WHE45950.1), *DcBBS-like* (XP 020690097), *BsCHS* (AHH25569.1), *PcCHS1* (AAX54693.1), and *VvSTS* (ABV82966) amino acid sequences, respectively (Figure 2). *D. officinale* DoBS1 retains the catalytic triad Cys164-His304-Asn337 characteristic of plant type III PKS, along with the highly conserved sequence G373FGPG (positioned according to the DoBS1 amino acid sequence). This suggests that DoBS1 and type III PKS may share similar functions. However, variations in the amino acid sequence could account for the structural and functional diversity observed among type III PKS. Several amino acid residues within the active center cavity, including Thr132, Ser133, Thr194, Thr197, Gly256, Phe265, and Ser338, are thought to play a critical role in determining both the length of the polyketide chain and the specificity for the initial substrate, as well as the final product [37,38]. In this investigation, the amino acid residues Thr197, Phe265, and Ser338 in the DoBS1 sequence were substituted with Leu, Leu, and Met (Figure 2). These substitutions may result in differences in substrate recognition between this sequence and others.

### 2.3. Phylogenetic Analysis

Four functionally characterized bacterial type III PKSs, namely *Pseudomonas fluorescens PhlD* (AAB48106), *Streptomyces griseus RppA* (BAA33495), *Mycobacterium tuberculosis PKS18* (A70958), and *Amycolatopsis mediterranei DpgA* (CAC48378), were selected as outgroups. The DoBS1 protein sequences were aligned using NCBI BLAST to screen type III PKSs amino acid sequences in the GenBank database.

**Figure 3:**
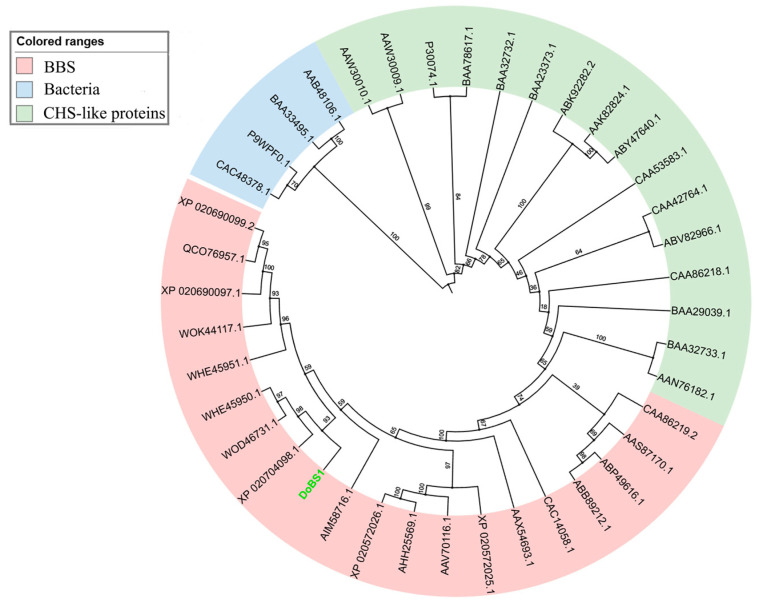
The phylogenetic relationship of type III PKS. The numbers at the nodes represent the bootstrap. Source data are provided in the Supplementary Material titled “Construct Phylogenetic Tree Source Files”.

Subsequently, a phylogenetic tree was constructed for 36 type III polyketide synthase (type III PKS) from 22 plant species (Figure 3). The analysis indicates that DoBS1 shares the highest homology with *D. catenatum* DcBBS (XP 020704098.1), *D. officinale* DoBBS-like protein (WOD46731.1), and *D. sinense* DsBBS1 (WHE45950.1). These results show that DoBS1 was clustered into the BBS group, which is deemed to have an important role in the biosynthesis of bibenzyl compounds.

### 2.4. Functional Expression of DoBS1 in E. coli, Purification

**Figure 4:**
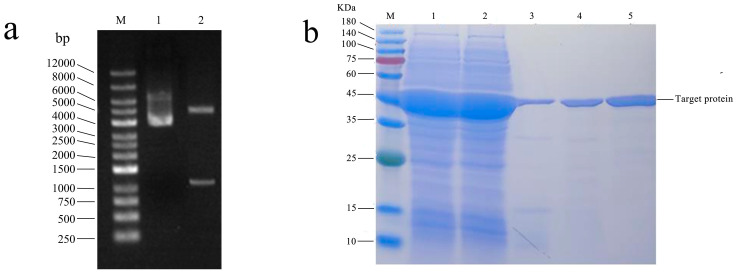
Figure of the pET-28a-DoBS1 recombinant plasmid construction and protein purification results. (**a**) Electrophoresis pattern of double-enzyme-digested recombinant plasmid. M: DNA marker (250–12,000 bp); Lane 1: results of double digestion of XbaI-XhoI empty vector; Lane 2: Results of double digestion of the pET-28a-DoBS1 recombinant plasmid. (**b**) DoBS1 was purified with the Ni^2+^-NTA column. M: 180 kDa Prestained Protein Marker; Lane 1: supernatant crude protein; Lane 2: the effluent obtained after incubating the supernatant crude protein with the equilibrated column packing for 1 h; Lane 3: equilibrate with 20 mM imidazole; Lane 4: washed with 50 mM imidazole; Lane 5: elution with 500 mM imidazole.

The target gene was integrated into the pET-28a expression vector (Figure S8). The plasmid was transformed to the *E. coli* BL21 (DE3) competent cells, positive clones were screened by colony PCR, and the plasmid was extracted for double enzyme digestion and sent to sequencing for verification, and the recombinant plasmid pET-28a-DoBS1 was obtained after digestion and verification (Figure 4a). The purified recombinant proteins were analyzed by SDS-PAGE with a relative molecular weight of 42.7 kDa, as shown in Figure 4b.

### 2.5. Confirmation Analysis of the Target Protein

**Figure 5:**
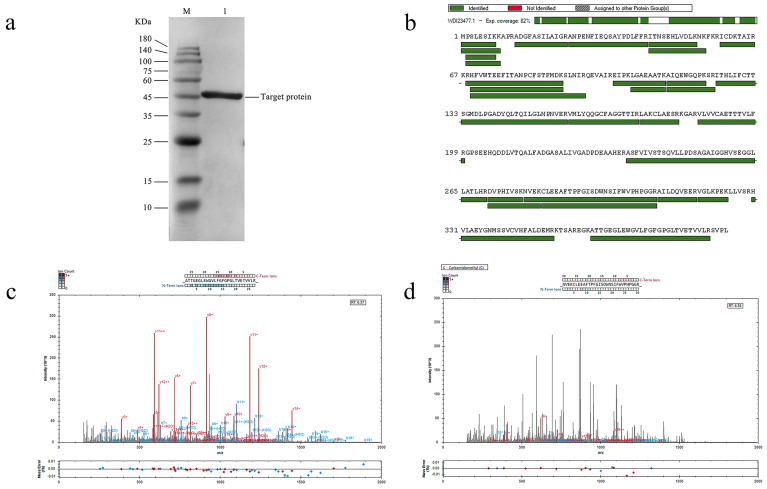
The results of the target protein verification. (**a**) Western blot analysis of the final puri-fied protein. M: 180 kDa predicted protein marker; Lane 1: Target protein. (**b**) The coverage of the identified peptides on the protein. (**c**) MS/MS analysis of peptide ion 1 (ATT-GEGLEWGVLFGFGPGLTVETVVLR). (**d**) MS/MS analysis of peptide ion 2 (NVEKCLEE-AFTPFGISDWNSIFWVPHPGGR).

A Western blot (WB) analysis was performed to confirm that the purified protein was indeed the target protein. As shown in Figure 5a, the WB analysis revealed a distinct band at the expected position, confirming the protein as DoBS1.

To enhance the accuracy of protein identification, additional mass spectrometry experiments were conducted. The DoBS1 protein, purified via SDS-PAGE, was subjected to trypsin hydrolysis and subsequently analyzed using mass spectrometry. Following the established fragmentation rules of protein mass spectrometry, a total of 29 peptide fragments were examined. Partial MS/MS spectra of these peptide fragments are presented in Figure 5c,d, while the remaining MS/MS spectra are provided in the Supplementary Material (Figures S9–S35). A comparison of these peptides with the protein sequences uploaded to NCBI (Accession no. WDI23477) reveals that the amino acid sequences of the 29 peptides were consistent with the sequence of the protein encoded by the *DoBS1* gene, achieving a coverage rate of 82.10% (Figure 5b). The calculated molecular weight of the protein was 42.7 kDa, which is consistent with the molecular weight results obtained from the SDS-PAGE analysis of DoBS1. This indicates that the identified protein corresponds to the target protein.

### 2.6. Enzyme Activity Analysis

**Figure 6:**
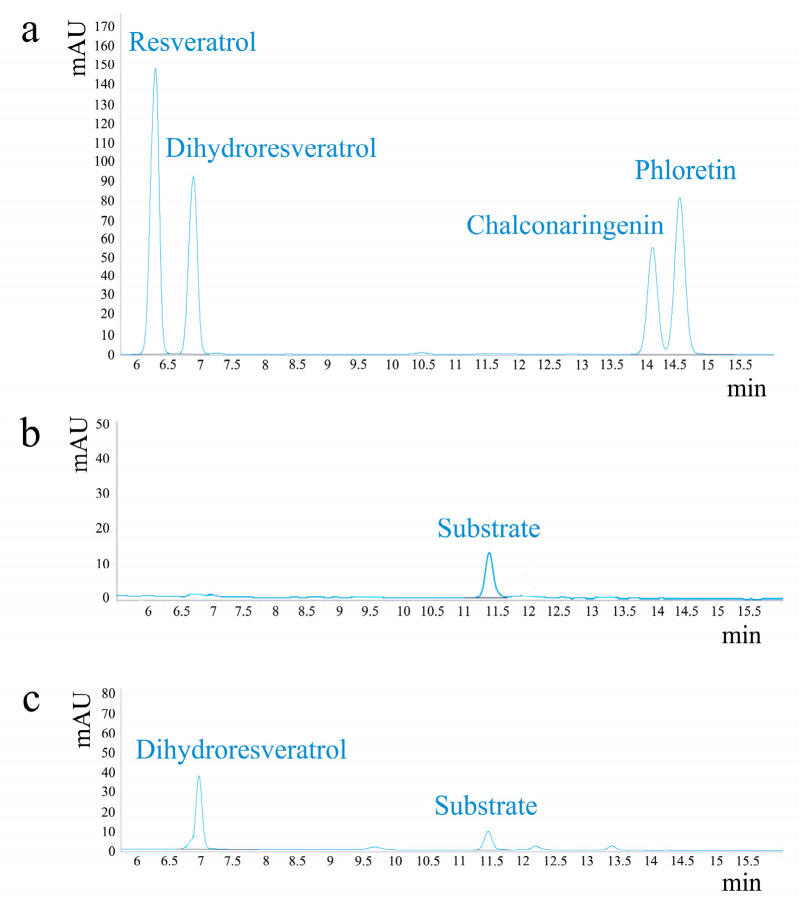
Analysis of the enzymatic reaction products of the DoBS1 protein. (**a**) HPLC chromatograms of the catalytic reaction standard mix. (**b**) Blank group. (**c**) HPLC chromatogram of the reaction product.

To investigate the enzymatic activity of the DoBS1 protein, a substrate was introduced for an in vitro enzymatic reaction with the target protein, and the formation of the desired product was detected by HPLC. For the phenylpropanoid pathway, the combination of one molecule of p-coumaroyl-CoA and three molecules of malonyl-CoA is a common substrate for the biosynthesis of flavonoids and bibenzyl. Dihydroresveratrol (DHR) is formed by the action of bibenzyl synthase. The retention times for the four standards were 6.293 min, 6.814 min, 14.150 min, and 14.609 min, respectively (Figure 6a). The signal peak occurred in the experimental group at 6.938 min (Figure 6c), which was similar to that of the DHR standard, while no signal peak occurred in the blank group (Figure 6b). It was concluded that the DoBS1 protein catalyzes the formation of DHR using 4-coumaroyl-CoA and 3-malonyl-CoA.

**Figure 7:**
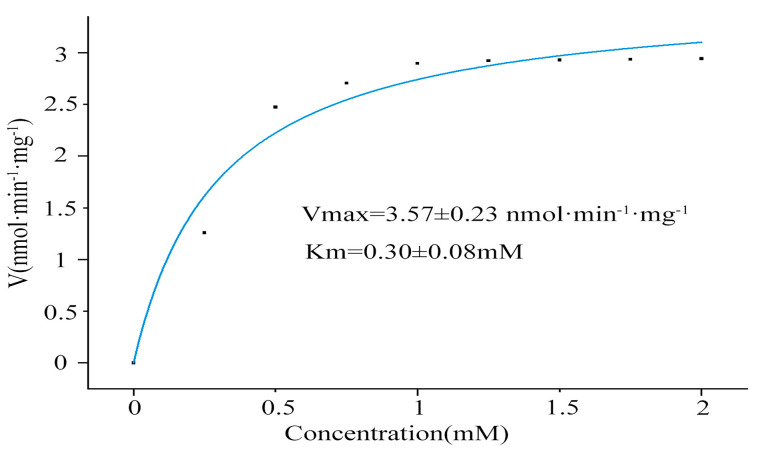
Kinetic analysis of DoBS1 toward p-coumaroyl-CoA. The Michaelis-Menten equation, expressed as V = Vmax[S]/(Km + [S]). The calculated values for Vmax and Km of the DoBS1 pro-tein were obtained. An adjusted R-squared value of 0.99787 indicates an excellent fit of the curve to the observed value.

The Vmax/Km of the recombinant DoBS1 protein for the dihydroresveratrol production were 3.57 ± 0.23 nmol·min^−1^·mg^−1^ and 0.30 ± 0.08 mmol (Figure 7).

## 3. Discussion

*D. officinale* has a rich history of medicinal utilization, as documented in the Chinese Pharmacopoeia (published in 2020). Ancient books, such as the *Shen Nong Ben Cao Jing*, extol the virtues of *Dendrobium* as a superior herbal medicine that fortifies “Yin” and invigorates the five viscera. *D. officinale* plays crucial pharmacological roles due to its rich content of polysaccharides, alkaloids, phenanthrenes, and bibenzyls [39]. DHR is a bibenzyl compound known for its diverse pharmacological activities, including anticancer and anti-inflammatory properties [40]. At present, the research on DHR mainly focuses on its pharmacological activity, but the synthetic pathway of DHR in *D. officinale* has not been elucidated. In this study, the *DoBS1* gene was cloned from *D. officinale*, and heterologous expression in *Escherichia coli* to obtain the corresponding protein, along with catalytic experiments, DHR was conducted to investigate the biosynthesis of bibenzyls.

Bibenzyl synthase belongs to the type III PKS gene family, which is known to be a homodimer with a protein size of 40 to 45 kDa [41]. Chen et al. identified the recombinant DsBBS-HisTag protein, which was constructed and expressed in *E. coli*, with a molecular weight of approximately 45 kDa [21]. Similarly, Liu et al. reported the recombinant DoBBS protein, also constructed and expressed in *E. coli*, with a molecular weight of approximately 40 kDa [35]. In contrast, our research indicates that the molecular weight of the DoBS1 protein is 42.7 kDa, which aligns with their findings. This matches the characteristics of the DoBS1 protein identified in *D. officinale*, indicating the discovery of a novel member of the BBS enzyme family.

In this study, the Vmax and Km of DoBS1 for DHR were 3.57 ± 0.23 nmol·min^−1^·mg^−1^ and 0.30 ± 0.08 mmol, respectively, realizing the catalytic functional activity of the enzyme protein. An enzyme activity analysis indicated that the recombinant DoBS1 protein could use 4-coumaryol-CoA and malonyl-CoA as substrates for DHR production in vitro. This finding is consistent with the known conjugated bienzyl synthase activity observed in orchid plants, such as *D. officinale* and *Phalaenopsis* [42,43,44]. Previous studies have indicated that bibenzyl synthases, which have been cloned, expressed, and purified from various plant sources, exhibit differing catalytic efficiencies. The *CsBBS2* gene was cloned and expressed from *Cannabis sativa*, subsequently transformed and expressed in *Escherichia coli*, leading to the purification of the corresponding protein. DHR was synthesized through the catalysis of dihydro-p-coumaroyl coenzyme A and malonyl coenzyme A, yielding a Km of 5.91 ± 3.95 µmol/L and a Kcat of 2.26 × 10^−3^ ± 5.46 × 10^−4^ s^−1^ [26]. Additionally, nine genes were cloned and expressed from *D. officinale*, demonstrating their capability to produce DHR using p-hydroxyphenylpropionyl and malonyl-CoA as substrates. Among these, DoBBS8 bibenzyl synthase exhibited the highest activity, with a Km of 61.83 µmol/L and a Kcat of 0.54 min^−1^ [35]. Furthermore, some researchers have cloned and expressed the *DsBBS* gene from *D. sinense*, which catalyzes p-coumaroyl coenzyme A and malonyl coenzyme A to produce resveratrol, resulting in a Vmax of 0.88 ± 0.07 pmol s^−1^ mg^−1^ [21]. The Vmax observed in our study is lower than that of *D. officinale* DoBBS8, but higher than that of *cannabis*. This discrepancy may be due to the different catalytic substrates used and the differences between species. The findings of this study suggest that, despite the use of a different substrate compared to *Cannabis sativa* and *D. officinale*, the final product remained DHR. Conversely, although the same substrate as *D. sinense* was employed, the resulting products differed. The mechanisms underlying these discrepancies warrant further investigation.

The amino acid sequence and structure of an enzyme protein determine the catalytic activity of the enzyme. By comparing the amino acid sequences with different plant type III PKSs, it was found that DoBS1 has a similar three-dimensional structure and catalytic mechanism to the plant type III PKS family members, and the amino acid sequences of DoBBS1-DoBBS9 studied by Liu et al. in this study were also compared, and the homology ranged from 69.39% to 96.67% (Figure S7). Amino acid residues Thr197, Phe265, and Ser338 located in the active cavity of these 10 amino acid sequences were found to be replaced by Leu, Leu, and Met, and it was speculated that the substitutions of key amino acids at these specific positions may determine the selectivity of different plant type III polyketide synthases for the starting substrate and the length of the polyketide chain extension, resulting in the formation of different products [37,38,45]. However, the catalytic rate in this study was lower than that of DoBBS8, due to the different substrates that were used, and a prominent reason may be the differences in the amino acid sequences with 96.67% homology—12 amino acid differences between DoBS1 and DoBBS8. To unravel the catalytic mechanism of benzylase biosynthesis, the 12 different amino acids will be focused on to decide the activity of catalytic efficiency.

## 4. Materials and Methods

### 4.1. Materials

#### 4.1.1. Plant Materials

*D. officinale* plants are conserved in the Dendrobium Germplasm Resource Bank of the Guizhou Academy of Forestry. Healthy leaves with uniform growth should be selected for sampling and stored in liquid nitrogen at −80 °C for future use.

#### 4.1.2. Chemicals/Kits/Equipment

Chemicals and reagents were purchased from Sigma Aldrich (St. Louis, MO, USA), Biodee Biotech Co., Ltd. (Beijing, China), Hangzhou Wahaha Group Co., Ltd. (Hangzhou, China), and Honeywell Burdick & Jackson Co., Ltd. (Beijing, China), unless otherwise noted. Primer synthesis and DNA sequencing were performed by Sangon Biotech Co., Ltd. (Shanghai, China). A Trizol extraction kit, column DNA gel recovery kit, restriction endonucleases NdeI, isopropyl-β-D-thiogalactopyranoside (IPTG) and DNA marker were purchased from Sangon Biotech Co., Ltd. (Shanghai, China). Ni-agarose gel 6FF was purchased from Solebao Technology Co., Ltd. (Beijing, China), and mouse anti-His antibody and goat anti-mouse IgG were purchased from Tiangen Biological Co., Ltd. (Beijing, China). Chemical standards for substrates, including resveratrol, dihydroresveratrol, phloretin, naringenin-chalcone malonyl-CoA, and p-coumaroyl-CoA, were purchased from McLean Biochemical Technology Co., Ltd. (Shanghai, China). A mass spectrometry analysis was completed by Majorbio Bio-pharm Technology Co., Ltd.(Shanghai, China). Enzyme activity analyses were performed on an Agilent 1260 Series HPLC system (Agilent Technologies, Palo Alto, CA, USA) equipped with a DAD detector.

### 4.2. Research Methods

#### 4.2.1. Cloning of Homologous BBS Gene Fragments

Genomic DNA was isolated from young leaves using a modified cetyltrimethylammonium bromide (CTAB) method [46]. Degenerate primers were generated based on the consensus degenerate hybrid oligonucleotide primers (CODEHOP) strategy using the Blockmaker program (https://bio.tools/blocks_www_server, URL(accessed on 3 December 2022)). The Degenerate primers employed are detailed in the Supplementary Material (Table S1). The PCR reaction protocol consisted of predenaturation at 94 °C for 2 min, denaturation at 94 °C for 30 s, annealing at 58 °C for 30 s, and extension at 72 °C for 1 min. The following 3 steps will carry out 35 repeated cycles, final extension at 72 °C for 5 min, and storage at 4 °C.

#### 4.2.2. Cloning of PKS cDNA

RNA was extracted from *D. officinale* according to the protocol of an RNA extraction kit, while its quality and concentration were assessed. A full-length cDNA was amplified using gene-specific primers based on the obtained open reading frame (ORF) sequence: 5′-GGGGGTTGACATGCCAGGT-3′ (RTDoBS1-F) and 5′-TCCGGCAATAAGACTTGAGATGTAG-3′ (RTDoBS1-R). The PCR program includes reverse transcription at 50 °C for 30 min, pre-denaturation at 94 °C for 2 min, denaturation at 94 °C for 30 s, annealing at 58 °C for 30 s, and extension at 72 °C for 1 min. The PCR reaction is run for 35 cycles, followed by a final extension at 72 °C for 5 min. The program is then stored at 4 °C. The gel-purified PCR sequence product was ligated into the pET-28a vector.

#### 4.2.3. Comparison of Homology Between DoBS1 Protein and Phylogenetic Tree Construction

The amino acid sequences of the encoded protein were compared using BLAST and DNAMAN [47]. Subsequently, the sequences were concatenated through a custom script, and a maximum likelihood tree was constructed using IQ-Tree (v2.2.0; settings: -m LG+F+R+C60-B 1000 -alrt 1000) [48]. Four bacterial type III PKSs were used as outgroups to root the tree. The resulting phylogenetic tree was then visualized using the iTOL v4 web server [49].

#### 4.2.4. Expression in *E. coli* and Purification of Recombinant Enzymes

The ORF of the cDNA was amplified using 5′ and 3′ PCR primers: sense, the XbaI site was underlined 5′-TCTAGAATGCCGAGCCTGGA-3′, and antisense, the XhoI site was underlined 5′-CTCGAGTTACAGCGGCACACTG-3′. The amplified DNA was digested with XbaI/XhoI and cloned into the XbaI/XhoI sites of pET-28a (Novagen, Darmstadt, Germany). The recombinant enzyme contained a hexahistidine tag at the C-terminus.

The recombinant plasmid pET-28a (+) was introduced into competent *E. coli BL21* (*DE3*) cells, followed by heat shock at 42 °C. The cells were then plated on a plate containing 30 μg/mL kanamycin and incubated overnight at 37 °C. A single clone colony was picked and inoculated into a liquid medium containing 30 µg/mL kanamycin and cultured at 37 °C overnight. It was then incubated at an absorbance at 600 nm (A600) of 0.6, then 0.5 mM isopropyl thio-β-d-galactoside (IPTG) was added to continue incubation for 6 h at 37 °C. Samples without inducers were used as negative controls. The cells were then centrifuged at 4000 rpm for 10 min, the supernatant discarded, and the cells harvested. Buffer A (1× PBS, pH 7.4) was added to the cells, followed by suspension and sonication for complete lysis. The supernatant was collected and precipitated by centrifugation. The resulting precipitate was dissolved in buffer B (8 M urea, 50 mM Tris-Hcl, 300 mM NaCl, pH 8.0). Both the unpurified supernatant and the precipitated protein were monitored by SDS-PAGE.

When the OD value reaches 0.6, the cells were cultured overnight at 20 °C for full-scale expression, and cultivated overnight at 20 °C for large-scale expression. The cells were collected by centrifugation and dissolved in buffer C (50 mM Tris, 300 mM NaCl, 0.1% Triton X-100, 0.2 mM PMSF, pH 8.0). The solution was then sonicated and centrifuged to collect the supernatant crude protein. The supernatant was filtered through a 0.45 μm filter and the crude protein was purified. The supernatant was passed through a column of Ni^2+^-NTA (Solarbio, Beijing, China) containing Ni^2+^ as an affinity ligand. Then, starting with 5 mL of Ni^2+^-NTA, the column was washed with 5 times the column bed volume of binding buffer (50 mM Tris, 300 mM NaCl, pH 8.0) to equilibrate. The crude protein was incubated with the equilibrated column packing for 1 h, the effluent was collected, the column was washed with binding buffer, then it was washed with wash buffer (50 mM Tris, 300mM NaCl, 20/50 mM imidazole, pH 8.0), and the effluent was collected. The elution buffer (50 mM Tris, 300 mM NaCl, 500 mM imidazole, pH 8.0) was used for elution, and the flow-out was collected. The crude protein and flow-out components were separated, processed, sampled, and the purification efficiency was assessed by SDS-PAGE. The purified components were dialyzed in a protein storage buffer (50 mM Tris, 300 mM NaCl, pH 8.0) [50]. After dialysis, the components were concentrated with PEG20000, filtered through a 0.45 μm membrane filter, aliquoted 1 mL per tube, and stored at −80 °C. The protein concentration was determined by the Bradford method using bovine serum albumin as standard.

#### 4.2.5. Western Blotting

A polyacrylamide gel was prepared with a loading amount of 1 μg. Following separation of the protein mixture by SDS-PAGE, the protein was transferred to a PVDF membrane using the ‘sandwich’ method with a membrane transfer instrument. Skimmed milk powder (5%) was utilized for blocking at 37 °C for a duration of 2 h with gentle agitation in a shaker. Subsequently, Hislabeled mouse monoclonal antibody (1:2000 dilution) was added and incubated at 37 °C for 1 h with slow shaking. After discarding the primary antibody, the membrane was washed 4 times with PBST, followed by the addition of HRP-labeled goat anti-mouse secondary antibody (1:5000 dilution) and another incubation at 37 °C for 1 h with slow shaking. The membrane was washed 4 times with PBST; TMB chromogenic solution was used, pictures were taken, and the necessary images were saved.

#### 4.2.6. Mass Spectrometric Analyses

Take 100 μg of the DoBS1 purified protein solution and add acetonitrile to remove water, then dry the mixture for 10 min. Next, add TCEP (10 mM) for reduction and incubate at 37 °C for 60 min. After cooling to room temperature, add IAM (40 mM) for alkylation and allow it to react for 40 min in the dark. Centrifuge the resulting sample for 20 min at 10,000 rpm, then collect the precipitate and dissolve it in 100 μL of 100 mM TEAB. Add mass spectrometry grade trypsin at a mass ratio of 1:50 (trypsin:protein) and digest overnight at 37 °C. Following digestion, quickly raise the temperature to 100 °C to terminate the reaction. Finally, pass the enzymatically hydrolyzed sample through a 0.22 μm microporous filter membrane and subject it to mass spectrometry detection and analysis (Thermo Fisher Scientific, San Jose, CA, USA).

The conditions for mass spectrometry detection and analysis are as follows: (1) HPLC conditions: use a uPAC High Throughput column (75 μm × 5.5 cm, Thermo, USA) with mobile phases A and B consisting of 0.1% formic acid and acetonitrile, respectively. The mobile phase gradient elution conditions are: 0–0.1 min, 4–8% mobile phase B; 0.1–1 min, 8–12.5% mobile phase B; 1–1.1 min, 12.5–12.6% mobile phase B; 1.1–3.1 min, 12.6–22.5% mobile phase B; 3.1–4.6 min, 22.5–45% mobile phase B; 4.6–5 min, 45–99% mobile phase B; and 5–7 min, 99% mobile phase B. The flow rate is 0.2 mL/min, and the loading volume is 5 μL. The samples separated by nanoliter high-performance liquid chromatography are analyzed by DIA mass spectrometry using a mass spectrometer. (2) Mass spectrometry conditions: operate in positive ion mode with the ion source voltage set to 1.5 kV. Both MS and MS/MS are detected and analyzed by the mass spectrometer, and data acquisition is performed in DIA mode [51].

The mass spectrometry scan range is set to 100–1700 *m/z*. Protein identification will be performed using Spectronaut 19.0 software with parameter settings of protein FDR ≤ 0.01, peptide FDR ≤ 0.01, peptide confidence ≥ 99% and XIC width ≤ 75 ppm.

#### 4.2.7. Enzymatic Assay and HPLC Analysis

To verify the activity of the recombinant protein, 50 µL volume of enzyme reaction containing 10 µg DoBS1-His tag fusion protein, 2.5 mM malonyl-CoA lithium salt and 2.5 mM 4-coumaroyl-CoA was incubated at 37 °C for 30 min. Finally, 50 µL of methanol was added to stop the reaction. After centrifugation at 12,000 rpm for 10 min, the samples were filtered through a 0.22 μm filter. The enzymatic products were analyzed by high-performance liquid chromatography (HPLC) on an Agilent ZORBAX Eclipse Plus C18 reverse-phase column (4.6 × 150 mm, 3.5 μm, Agilent, CA, USA). The eluents were 0.1% phosphoric acid water (A) and acetonitrile (B) at a flow rate of 1.0 mL per 1 min. The following gradients were used: 0–8 min, 25% B; 8–9 min, 25–28% B; 9–18 min, 28% B; 18–19 min, 28–25% B; 19–25 min, 25% B. The detection wavelength was 220 nm, the column temperature was 40 °C, and the injection volume was 8 μL. A standard solution of reference compounds was used for quantification, which includes Chalconaringenin, Phloretin, Resveratrol, and Dihyroresveratrol.

## 5. Conclusions

In this study, the gene *DoBS1* was successfully cloned from *D. officinale*. Phylogenetic analyses showed that *DoBS1* belongs to the BBS family. The *DoBS1* was heterologously expressed in *Escherichia coli* and garnered in a protein with a molecular weight of 42.7 kDa. The enzyme protein catalyzed the production of DHR from 4-coumaroyl-CoA and 3 malonyl-CoA in vitro. The Vmax and Km of the recombinant protein for DHR were 3.57 ± 0.23 nmol·min^−1^·mg^−1^ and 0.30 ± 0.08 mmol, respectively. The study successfully isolated and identified a novel BBS in *D. officinale*, which could contribute to a better understanding of the active site of bibenzyl biosynthesis.

## Data Availability

The gene sequence and protein sequences for *Dendrobium officinale* are deposited in the NCBI database (https://www.ncbi.nlm.nih.gov/; Accession No.: WDI23477).

## Supplementary Materials

The following supporting information can be downloaded at: https://www.mdpi.com/article/10.3390/molecules29225320/s1, Figure S1: Homology comparison result of the 22st gene fragment in Table S2, Figure S2: Homology comparison result of the 26st gene fragment in Table S2, Figure S3: Homology comparison result of the 28st gene fragment in Table S2; Figure S4: Electrophoresis of full-length PCR product of DoBS1 gene cloning, Figure S5: Results of DoBS1 gene sequencing, Figure S6: The DoBS1 gene coding sequence and amino acids, Figure S7: Comparison of amino acid sequences of BBS derived from the same plant (*D. officinale*), Figure S8: Construction of DoBS1 into PET-28a vector, Figure S9: MS /MS analysis of peptide ion 3 (ADGFASILAIGR); Figure S10: MS /MS analysis of peptide ion 4 (AILDQVEER), Figure S11: MS /MS analysis of peptide ion 5 (AIQEWGQPK), Figure S12: MS /MS analysis of peptide ion 6 (AIQEWGQPKSR), Figure S13: MS /MS analysis of peptide ion 7 (ANPENFIEQSAYPDLFFR, Figure S14: MS /MS analysis of peptide ion 8 (ASFVIVSTSQVLLPDSAGAIGGHVSEGGLLATLHR), Figure S15: MS /MS analysis of peptide ion 9 (CLEEAFTPFGISDWWSIFWVPHPGGR), Figure S16: MS /MS analysis of peptide ion 10 (DVPHIVSK), Figure S17: MS /MS analysis of peptide ion 11(DVPHIVSKNVEK), Figure S18: MS /MS analysis of peptide ion 12 (EIPKLGAEAATK), Figure S19: MS /MS analysis of peptide ion 13 (HFVWTEEFITANPCFSTFMDK), Figure S20: MS /MS analysis of peptide ion 14 (HVLAEYGNMSSVCVHFALDEMR), Figure S21: MS /MS analysis of peptide ion 15 (ICDKTAIR); Figure S22: MS /MS analysis of peptide ion 16 (ITHLIFCTTSGMDLPGADYQLTQILGLNPNVER), Figure S23: MS /MS analysis of peptide ion 17 (ITNSEHLVDLK), Figure S24: MS /MS analysis of peptide ion 18 (ITNSEHLVDLKNK); Figure S25: MS /MS analysis of peptide ion 19 (LAKCLAESR), Figure S26: MS /MS analysis of peptide ion 20 (LGAEAATK), Figure S27: MS /MS analysis of peptide ion 21 (MPSLESIK), Figure S28: MS /MS analysis of peptide ion 22 (PSLESIK), Figure S29: MS /MS analysis of peptide ion 23 (PSLESIKK), Figure S30: MS /MS analysis of peptide ion 24 (RHFVWTEEFITANPCFSTFMDK), Figure S31: MS /MS analysis of peptide ion 25 (VGLKPEK), Figure S32: MS /MS analysis of peptide ion 26 (VLVVCAETTTVLFR), Figure S33: MS /MS analysis of peptide ion 27 (VMLYQQGCFAGGTTIR), Figure S34: MS /MS analysis of peptide ion 28 (ATTGEGLEWGVLFGFGPGLTVETVVLR), Figure S35: MS /MS analysis of peptide ion 29 (NVEKCLEEAFTPFGISDWWSIFWVPHPGGR), Figure S36: Original Western Blot image; Table S1: The table of homemade degenerate primer combination information, Table S2: Table of specific fragments amplified by degenerate primers; Supplementary material: Construct phylogenetic tree source files.
