# Supplementary material for: Biological Characteristics of a Novel Bibenzyl Synthase (DoBS1) Gene from Dendrobium officinale Catalyzing Dihydroresveratrol Synthesis
Source: Molecules. 2024 Nov 12;29(22):5320. doi: 10.3390/molecules29225320 (PMC11596957; doi:10.3390/molecules29225320)
Supplement: Supplementary file 1 [file molecules-29-05320-s001.zip › molecules-3220024-supplementary.pdf]

Supporting Information

Biological Characteristics of a Novel Bibenzyl Synthase（DoBS1） Gene from Dendrobium officinale Catalysing Dihydroresveratrol Synthesis

Table of Contents

Supplementary Table...S2
Table S1. The table of homemade degenerate primer combination information...S2
Table S2. Table of specific fragments amplified by degenerate primers...S3
Supplementary Figures...S5
Figure S1. Homology comparison result of the 22st gene fragment in Table S2...S5
Figure S2. Homology comparison result of the 26st gene fragment in Table S2...S5
Figure S3. Homology comparison result of the 28st gene fragment in Table S2...S6
Figure S4. Electrophoresis of full-length PCR product of DoBS1 gene cloning...S12
Figure S5. Results of DoBS1 gene sequencing...S7
Figure S6. The DoBS1 gene coding sequence and amino acids...S7
Figure S7.Comparison of amino acid sequences of BBS derived from the same plant (*D.officinale*)...S8
Figure S8. Construction of DoBS1 into PET-28a vector...S9
Figure S9. MS /MS analysis of peptide ion 3 (ADGFASILAIGR)...S9
Figure S10. MS /MS analysis of peptide ion 4 (AILDQVEER) ...S9
Figure S11. MS /MS analysis of peptide ion 5 (AIQEWGQPK) ...S10
Figure S12. MS /MS analysis of peptide ion 6 (AIQEWGQPKSR) ...S10
Figure S13. MS /MS analysis of peptide ion 7 (ANPENFIEQSAYPDLFFR...S10
Figure S14. MS /MS analysis of peptide ion 8 (ASFVIVSTSQVLLPDSAGAIGGHVSEGGLLATLHR) ...S11
Figure S15. MS /MS analysis of peptide ion 9 (CLEEAFTPFGISDWWSIFWVPHPGGR)...S11
Figure S16. MS /MS analysis of peptide ion 10 (DVPHIVSK)...S11
Figure S17. MS /MS analysis of peptide ion 11(DVPHIVSKNVEK)...S12
Figure S18. MS /MS analysis of peptide ion 12 (EIPKLGAEAATK)...S12
Figure S19. MS /MS analysis of peptide ion 13 (HFVWTEEFITANPCFSTFMDK)...S12
Figure S20. MS /MS analysis of peptide ion 14 (HVLAEYGNMSSVCVHFALDEMR)... S13
Figure S21. MS /MS analysis of peptide ion 15 (ICDKTAIR) ...S13
Figure S22. MS /MS analysis of peptide ion 16 (ITHLIFCTTSGMDLPGADYQLTQILGLNPNVER) ...S13
Figure S23. MS /MS analysis of peptide ion 17 (ITNSEHLVDLK)... S14
Figure S24. MS /MS analysis of peptide ion 18 (ITNSEHLVDLKNK)... S14
Figure S25. MS /MS analysis of peptide ion 19 (LAKCLAESR)... S14
Figure S26. MS /MS analysis of peptide ion 20 (LGAEAATK) ... S15
Figure S27. MS /MS analysis of peptide ion 21 (MPSLESIK). ... S15
Figure S28. MS /MS analysis of peptide ion 22 (PSLESIK)... S15
Figure S29. MS /MS analysis of peptide ion 23 (PSLESIKK)...S16
Figure S30. MS /MS analysis of peptide ion 24 (RHFVWTEEFITANPCFSTFMDK)...S16
Figure S31. MS /MS analysis of peptide ion 25 (VGLKPEK)... S16
Figure S32. MS /MS analysis of peptide ion 26 (VLVVCAETTTVLFR) ...S17
Figure S33. MS /MS analysis of peptide ion 27 (VMLYQQGCFAGGTTIR)...S17
Figure S34. MS /MS analysis of peptide ion 28 (ATTGEGLEWGVLFGFGPGLTVETVVLR) ...S17
Figure S35. MS /MS analysis of peptide ion 29 (NVEKCLEEAFTPFGISDWWSIFWVPHPGGR)... S18
Figure S36. Original Western Blot image... S18
Supplementary Material...S19
Construct phylogenetic tree source files...S19

Supplementary Table

Table S1. The table of homemade degenerate primer combination information. Specific information on degenerate primers used in method 4.2.1.

Number	Primer combination	Primer type	Sequence orientation	Sequences	UP/DN
T1	P39	degenerate primer	5`-3`	ACT TCT ggC gTT gAT TAg CCN ggN gCN gA	UP
	P41	degenerate primer	5`-3`	AAA gAA CgC AAg CAg AAg ACA TRT TNC CRT A	DN
T2	P39	degenerate primer	5`-3`	ACT TCT ggC gTT gAT TAg CCN ggN gCN gA	UP
	P43	degenerate primer	5`-3`	TCC ATT TTC Tgg ATC gCT CAY CCN ggN gg	DN
T3	P39	degenerate primer	5`-3`	ACT TCT ggC gTT gAT TAg CCN ggN gCN gA	UP
	P45	degenerate primer	5`-3`	CTT TTC CTg CgT TCg TCT TCT gTA YAA NAg YAT	DN
T4	P40	degenerate primer	5`-3`	TgA gAC Tgg CTA Agg ATT TTg CNg ARA AYA A	UP
	P41	degenerate primer	5`-3`	AAA gAA CgC AAg CAg AAg ACA TRT TNC CRT A	DN
T5	P40	degenerate primer	5`-3`	TgA gAC Tgg CTA Agg ATT TTg CNg ARA AYA A	UP
	P43	degenerate primer	5`-3`	TCC ATT TTC Tgg ATC gCT CAY CCN ggN gg	DN
T6	P40	degenerate primer	5`-3`	TgA gAC Tgg CTA Agg ATT TTg CNg ARA AYA A	UP
	P45	degenerate primer	5`-3`	CTT TTC CTg CgT TCg TCT TCT gTA YAA NAg YAT	DN
T7	P42	degenerate primer	5`-3`	ggA ggC TgC TgT TAA ggC TAT CVM NgA RTg ggg	UP
	P41	degenerate primer	5`-3`	AAA gAA CgC AAg CAg AAg ACA TRT TNC CRT A	DN
T8	P42	degenerate primer	5`-3`	ggA ggC TgC TgT TAA ggC TAT CVM NgA RTg ggg	UP
	P43	degenerate primer	5`-3`	TCC ATT TTC Tgg ATC gCT CAY CCN ggN gg	DN
T9	P42	degenerate primer	5`-3`	ggA ggC TgC TgT TAA ggC TAT CVM NgA RTg ggg	UP
	P45	degenerate primer	5`-3`	CTT TTC CTg CgT TCg TCT TCT gTA YAA NAg YAT	DN
T10	P44	degenerate	5`-3`	gAA gCT gCT ACT AAg gCT ATT AAR gAR	UP

		primer		Tgg gA	
T11	P41	degenerate primer	5`-3`	AAA gAA CgC AAg CAg AAg ACA TRT TNC CRT A	DN
	P44	degenerate primer	5`-3`	gAA gCT gCT ACT AAg gCT ATT AAR gAR Tgg gA	UP
	P43	degenerate primer	5`-3`	TCC ATT TTC Tgg ATC gCT CAY CCN ggN gg	DN
T12	P44	degenerate primer	5`-3`	gAA gCT gCT ACT AAg gCT ATT AAR gAR Tgg gA	UP
	P45	degenerate primer	5`-3`	CTT TTC CTg CgT TCg TCT TCT gTA YAA NAg YAT	DN
T13	P18	general primer	5`-3`	ggg gTT gAC ATg CCA ggT	UP
	P19	general primer	5`-3`	TCC AAA CAg AAC gCC CCA	DN

Table S2 Table of specific fragments amplified by degenerate primers

Number	Gene fragment sequence amplified by degenerate primers
1	GCTTTGCCGTGCCAGTCGAGCTATGTATTCATCCATGACTAATGACTGCCATAAGTAATCTAGCCAGAAGTA
2	GGTCTAAATTTCTAGATCTGCTCAGGCTATCACGCCAGAAGTAA
3	GCGTTGACGGCGTTCAGTCGAGTATGTCAGCACGATGCTAATCAACGCCAAAAGTAA
4	CTGGCTTGGTTCTGTGTACCGTCGCCAGATGTACAACGCCAGAAGTA
5	CCCTTATTTCTCGATGCCTTAAAGCAGCCCACGTGGAATGGGGTCGTCTTCAG
6	ACGACACAAATGACAGATCGGATCCTGGGCGGAAGTTATTGATGTTCCGGCGCGGTCGCCACATAGGACCCCCCGGGTTAAGCAATCCGAAAAAGGAAA
7	GGGGCGAATCGTATACGTTATAGTAAAAGCTACCAATTTCTAAATAAGGCACGTAGTGTCCCATGAACCACTTTTGCAAATTGGACTGGGCTACCCGACACTGCTTAACGAACCTTCCTTCTCGACCTTCCTGATCCAACCTGACCATCCGACTAGCTAGCTGGATCTGGTTGACCAAAGCCCCCTTCCTGGATAGCCTTAACAGCACCCTCCAAGGTG
8	ATTGGAACGGGCTTTCGTCTGATGCTCAGCTAGCTAGTCGGATGGTCAGGTCGGATCAGGAAGGTCGAGAAGGAAGGTTCGCTAAGCAGTGTCGGGTAGCCCAGTCCAATCTGCAAAAGTGGTTCATGGGACACTACGTGCCTTATCTAGAAATTGTGTAGCTCTGACTAGTAAGTGAGACGACTGCCCCCCCGGGTGGACGATCCAAAAAAAGGGAAA
9	GCCGGGGGCCGGTACACGTTAAAGTAGAGCTACTCAATTTCTAAATAAGGCACGTAGTGTCCCATGAACCACTTTTGCAAATTGGACTGGGCTACCCGACACTGCTTAACGAACCTTCCTTCTCGACCTTCCTGATCCAACCTGACCATCCGACTAACTAGCTGGATCTGGTTGACCAAAGCCCCCTTCCTGGATAGCCTTAACAGCAGCCTCCAAG
10	CCGGGCCAAAATATCGAGCACTAGTCGCATCCTCACTCATCTTCTGCACCACGAGCGGCATGGACTTACCTGGTGCTGACTATCAGCTCAACCCAACTTCTTGGCC

	TCAACCCTAATGTCCAGCGTGTCATGCTCTATCAGCAGGGTTGTTTTGCTGGCGGAACCACGCTTCGTCTCGCTAAGTGCCTTGCCAAAAGCCCCACGGGCGCACGTGTTCTTGTGGTTTGTGCGAAAACCCCCACTGTGCTATTTCCGGGACCGTCTAAAGACCTCCAAAACCATCTCGCGACCCAAGCTTTATTTGCTGATGGTGCCTCCGCACTTATAGGGGGGGCCGATCCACATGAAGCGGCCCGTGAGAGGGCCAGTTTCTTCTTACTCTCTACATCTCAAGTCTTATTGCCGGACTCTGCTGGTGCTATTGGAGATCATGTAAGCGAGGGAGGCCTCTTCCCCACGCTCCATAGAGATGTTCCAATTATTGTTACTAATAATGATAAAAGGTGGTTGGAAGATGTCCTCACCCCATTTATTATTACCGGTTGTAACACTTTCTGGAGGGCGCCGCCGGCCGGGGGTCCAATTCTCGTCCATGAGGAAGAGGAGATTGGGCTAGACGGAAATAAGCTCGCGGCCTCAAGGCGTGTGCTTTCTGACTACGGAAACATGCTCCTGCTT
11	GTGGCTGGGGCGACTATCCAGGCTCCCTACTACATCTTCTGTACACGAGCGGCATGGACTTACCTGGTGCTGACTATCACTCAATCACCTTCTTGGCCTCAACCCTTATGTCAACCGTGTCATGCTCTATCACAAGGGTTGTTTTGCTGCGGGAACCACTCTTCGTCTCCCTAAGACCCTTGCCAAAAGCCCCCCGGGCGCACTTGTTTCTTGTGGCTTGTGCGAAAACCCCCCCTGTGCTATTCCCGGGACCGTCTAAACACCTCCAAAATCATCTGGCGACCCAAGCTTTATTTGCTGATGGTGCCTCCGCACTTTTGTTGGGGGCCGATCTGATGAAATCTGGATGAGAGGGCCAGTTTCTTCTTTCTCTCTACATCTCAATACTTATTGCCGGACTCTGCCGGTGCTATTGGAGGCCATGTAAACGAGGGAGGCCTCTTCCCCACCCTCCATAAAGATGTTCCAATTATTGTTTCTAATAATGATAAAAGGTGGTTGGAAGATTCCTTCCCCCCTTTTATTCTTAACGATTATAACACTTTCTGCAGGGCGCCGCCTGCTGGGGCACCAACCCTCCTCCATGATGAGGAGGAGAGAGTGGTATAGAGACATAAGCTCTCGGTCGCTTCGAGTGTGCTTCCTGACTACGACAGCATGTTGTCTGCTGGTTTGTTATTTAATTA
12	CCCCCACCTATCTTTCACCCACATATTCCGCATCACTCACTCATCTTCTGCACCACGAGCGGCATGGACTTACCTGGTGCTGACTATCAGCTCACCCAACTCCTTGGCCTCAACCCAAATGTCGAGCGTGTCATGCTCTATCAGCAGGGTTGTTTCGCTGGCGGAACCACGCTCCGCCTGCCGAACCGCCCTCCGAGAACCACGCCGGCTTGCGTGAGCTTCTGGGTGTTCGAAATCACCGGGTTTGCGTTTCGCGGGACCGTGTGAACTCCTCTGGATTCTCTGGTCAACACCACACTGACTTTGTGACCCTGCCTTTGTTGTTTGATGGGGCGCTGATCCTGATCCCGGGGGCCACCGTCCATTGTTCCAACTTATATCAGGCCCAGTTTCTATACTCCCGGAATCCTCAGGTGGTATTGACGGACACCGTGGGGGCGAGGGTCTCCCCTTCCATCTCCTGAAAGACGTTGTCCCACTTATTTCTAAAAATATTGAGAAAAGTTGGTTGGAAGATTTCAAACCGCTTGGGAGACTTGACTATAATTTTTTTTTTTGCAGTGTGCATCCGGGCGGGCCAGCTGCCCTCCACCAGGGGGAAAAACAGACTGGGCTATACAGAGAAGAGCTCGCGGTCGAATCGAGTGTGCTTTCTGACTACTACAACATGACTTCTGCTTGCTTTTTATTTATTACTTCTGC

Supplementary Figures

select all 64 sequences selected | GenBank | Graphics | Distance tree of results | MSA Viewer

	Description	Scientific Name	Max Score	Total Score	Query Cover	E value	Per. Ident	Acc. Len	Accession
☑	Dendrobium officinale bibenzyl synthase-like protein mRNA, complete cds	Dendrobium officinale	689	689	77%	0.0	90.34%	1173	MH992135.1
☑	PREDICTED: Dendrobium catenatum bibenzyl synthase-like (LOC110105072), mRNA	Dendrobium catenatum	684	684	77%	0.0	90.15%	1283	XM_020834438.2
☑	Dendrobium officinale bibenzyl synthase-like protein mRNA, complete cds	Dendrobium officinale	664	664	74%	0.0	90.35%	1173	MH992136.1
☑	PREDICTED: Dendrobium catenatum bibenzyl synthase-like (LOC110105073), mRNA	Dendrobium catenatum	647	647	74%	2e-180	89.76%	1457	XM_020834440.2
☑	Dendrobium hercoglossum De345 DNA, similar to bibenzyl synthase, clone: De345C2	Dendrobium hercoglossum	636	636	74%	4e-177	89.37%	1016	LC771164.1
☑	Dendrobium officinale isolate P44 bibenzyl synthase-like protein mRNA, complete cds	Dendrobium officinale	634	634	74%	1e-176	89.35%	1173	OR727875.1
☑	Dendrobium hercoglossum De345 DNA, similar to bibenzyl synthase, clone: De345C3	Dendrobium hercoglossum	612	612	74%	7e-170	88.69%	1034	LC771165.1
☑	Dendrobium nobile De286 DNA, similar to bibenzyl synthase, clone: De286C12	Dendrobium nobile	610	610	74%	2e-169	88.54%	1034	LC771154.1
☑	Dendrobium thyrsiflorum De380 DNA, similar to bibenzyl synthase, clone: De380C4	Dendrobium thyrsiflorum	604	604	74%	1e-167	88.21%	1034	LC771155.1
☑	Dendrobium densiflorum De395 DNA, similar to bibenzyl synthase, clone: De395C5	Dendrobium densiflorum	593	593	74%	2e-164	87.82%	1032	LC771157.1
☑	Dendrobium densiflorum De395 DNA, similar to bibenzyl synthase, clone: De395C1	Dendrobium densiflorum	593	593	74%	2e-164	87.82%	902	LC771156.1
☑	Dendrobium sinense bibenzyl synthase 2 mRNA, complete cds	Dendrobium sinense	592	592	74%	9e-164	87.92%	1437	OP887150.1
☑	Dendrobium officinale isolate TPSH-1 BS1 (BS1) mRNA, complete cds	Dendrobium officinale	575	575	74%	9e-159	87.20%	1173	OQ458714.1
☑	Dendrobium fimbriatum De351 DNA, similar to bibenzyl synthase, clone: De351C1	Dendrobium fimbriatum	571	571	74%	1e-157	87.03%	1033	LC771160.1
☑	Dendrobium nobile isolate R2-P44 bibenzyl synthase-like protein mRNA, complete cds	Dendrobium nobile	571	571	74%	1e-157	86.61%	1173	OR687304.1
☑	Dendrobium kingianum De134 DNA, similar to bibenzyl synthase, clone: De134C6	Dendrobium kingianum	547	547	74%	2e-150	86.22%	804	LC771172.1
☑	Dendrobium macrophyllum De451 DNA, similar to bibenzyl synthase, clone: De451C21	Dendrobium macrophyllum	536	536	74%	4e-147	85.83%	768	LC771171.1
☑	Dendrobium speciosum De343 DNA, similar to bibenzyl synthase, clone: De343C11	Dendrobium speciosum	536	536	74%	4e-147	85.83%	962	LC771170.1
☑	Dendrobium spectabile De324 DNA, similar to bibenzyl synthase, clone: De324C2	Dendrobium spectabile	531	531	74%	2e-145	85.74%	1034	LC771167.1
☑	Dendrobium officinale bibenzyl synthase mRNA, complete cds	Dendrobium officinale	497	497	77%	2e-135	83.93%	1173	MH992134.1
☑	PREDICTED: Dendrobium catenatum bibenzyl synthase (LOC110115249), mRNA	Dendrobium catenatum	497	497	77%	2e-135	83.93%	1472	XM_020848439.2
☑	Dendrobium officinale bibenzyl synthase 8 mRNA, complete cds	Dendrobium officinale	486	486	74%	4e-132	84.25%	1173	OM809168.1
☑	Dendrobium amboinense De344 DNA, similar to bibenzyl synthase, clone: De344C26	Dendrobium amboinense	470	470	73%	4e-127	83.73%	1023	LC771169.1
☑	Dendrobium amboinense De344 DNA, similar to bibenzyl synthase, clone: De344C35	Dendrobium amboinense	448	448	73%	2e-120	82.94%	1021	LC771168.1

Figure S1. Homology comparison result of the 10st gene fragment in Table S2.

select all 37 sequences selected | GenBank | Graphics | Distance tree of results | MSA Viewer

	Description	Scientific Name	Max Score	Total Score	Query Cover	E value	Per. Ident	Acc. Len	Accession
☑	Dendrobium officinale bibenzyl synthase-like protein mRNA, complete cds	Dendrobium officinale	470	470	71%	4e-127	84.04%	1173	MH992135.1
☑	Dendrobium officinale bibenzyl synthase-like protein mRNA, complete cds	Dendrobium officinale	464	464	71%	2e-125	83.84%	1173	MH992136.1
☑	PREDICTED: Dendrobium catenatum bibenzyl synthase-like (LOC110105072), mRNA	Dendrobium catenatum	464	464	71%	2e-125	83.84%	1283	XM_020834438.2
☑	PREDICTED: Dendrobium catenatum bibenzyl synthase-like (LOC110105073), mRNA	Dendrobium catenatum	448	448	71%	2e-120	83.23%	1457	XM_020834440.2
☑	Dendrobium officinale isolate P44 bibenzyl synthase-like protein mRNA, complete cds	Dendrobium officinale	420	420	71%	4e-112	82.22%	1173	OR727875.1
☑	Dendrobium hercoglossum De345 DNA, similar to bibenzyl synthase, clone: De345C2	Dendrobium hercoglossum	412	412	69%	7e-110	82.46%	1016	LC771164.1
☑	Dendrobium nobile De286 DNA, similar to bibenzyl synthase, clone: De286C12	Dendrobium nobile	412	412	69%	7e-110	82.46%	1034	LC771154.1
☑	Dendrobium hercoglossum De345 DNA, similar to bibenzyl synthase, clone: De345C3	Dendrobium hercoglossum	401	401	69%	2e-106	82.01%	1034	LC771165.1
☑	Dendrobium thyrsiflorum De380 DNA, similar to bibenzyl synthase, clone: De380C4	Dendrobium thyrsiflorum	398	398	71%	2e-105	81.41%	1034	LC771155.1
☑	Dendrobium sinense bibenzyl synthase 2 mRNA, complete cds	Dendrobium sinense	398	398	71%	2e-105	81.41%	1437	OP887150.1
☑	Dendrobium densiflorum De395 DNA, similar to bibenzyl synthase, clone: De395C5	Dendrobium densiflorum	396	396	69%	7e-105	81.84%	1032	LC771157.1
☑	Dendrobium densiflorum De395 DNA, similar to bibenzyl synthase, clone: De395C1	Dendrobium densiflorum	396	396	69%	7e-105	81.84%	902	LC771156.1
☑	Dendrobium kingianum De134 DNA, similar to bibenzyl synthase, clone: De134C6	Dendrobium kingianum	370	370	71%	5e-97	80.40%	804	LC771172.1
☑	Dendrobium speciosum De343 DNA, similar to bibenzyl synthase, clone: De343C11	Dendrobium speciosum	364	364	71%	2e-95	80.20%	962	LC771170.1
☑	Dendrobium fimbriatum De351 DNA, similar to bibenzyl synthase, clone: De351C1	Dendrobium fimbriatum	361	361	68%	3e-94	80.54%	1033	LC771160.1
☑	Dendrobium macrophyllum De451 DNA, similar to bibenzyl synthase, clone: De451C21	Dendrobium macrophyllum	359	359	71%	1e-93	80.00%	768	LC771171.1
☑	Dendrobium spectabile De324 DNA, similar to bibenzyl synthase, clone: De324C2	Dendrobium spectabile	348	348	71%	2e-90	79.60%	1034	LC771167.1
☑	Dendrobium amboinense De344 DNA, similar to bibenzyl synthase, clone: De344C26	Dendrobium amboinense	285	285	69%	2e-71	77.66%	1023	LC771169.1
☑	Phalaenopsis sp. 'True Lady' chalcone synthase homolog mRNA, complete cds	Phalaenopsis hybrid cultivar	272	272	66%	1e-67	77.59%	1479	U88077.1
☑	Dendrobium amboinense De344 DNA, similar to bibenzyl synthase, clone: De344C35	Dendrobium amboinense	267	267	67%	6e-66	77.20%	1021	LC771168.1
☑	Phalaenopsis sp. 'pSPORT1' BibSy811 mRNA for bibenzyl synthase	Phalaenopsis hybrid cultivar	261	261	66%	3e-64	77.11%	1525	X79904.1

Figure S2. Homology comparison result of the 11st gene fragment in Table S2.

select all 58 sequences selected GenBank Graphics Distance tree of results MSA Viewer

Description	Scientific Name	Max Score	Total Score	Query Cover	E value	Per. Ident	Acc. Len	Accession
PREDICTED: Dendrobium catenatum bibenzyl synthase-like (LOC110105073), mRNA	Dendrobium ...	254	254	21%	5e-62	97.33%	1457	XM_020834440.2
Dendrobium thyrsiflorum De380 DNA, similar to bibenzyl synthase, clone: De380C4	Dendrobium t...	252	252	21%	2e-61	97.32%	1034	LC771155.1
PREDICTED: Dendrobium catenatum bibenzyl synthase-like (LOC110105072), mRNA	Dendrobium ...	248	248	20%	2e-60	97.28%	1283	XM_020834438.2
Dendrobium hercoglossum De345 DNA, similar to bibenzyl synthase, clone: De345C2	Dendrobium ...	248	248	21%	2e-60	96.67%	1016	LC771164.1
Dendrobium densiflorum De395 DNA, similar to bibenzyl synthase, clone: De395C5	Dendrobium ...	246	246	21%	8e-60	96.64%	1032	LC771157.1
Dendrobium densiflorum De395 DNA, similar to bibenzyl synthase, clone: De395C1	Dendrobium ...	246	246	21%	8e-60	96.64%	902	LC771156.1
Dendrobium officinale bibenzyl synthase-like protein mRNA, complete cds	Dendrobium ...	243	243	20%	1e-58	96.60%	1173	MH992136.1
Dendrobium officinale bibenzyl synthase-like protein mRNA, complete cds	Dendrobium ...	243	243	20%	1e-58	96.60%	1173	MH992135.1
Dendrobium sinense bibenzyl synthase 2 mRNA, complete cds	Dendrobium ...	241	241	21%	4e-58	95.97%	1437	OP887150.1
Dendrobium macrophyllum De451 DNA, similar to bibenzyl synthase, clone: De451C21	Dendrobium ...	237	237	21%	5e-57	94.77%	768	LC771171.1
Dendrobium hercoglossum De345 DNA, similar to bibenzyl synthase, clone: De345C3	Dendrobium ...	237	237	21%	5e-57	95.33%	1034	LC771165.1
Dendrobium spectabile De324 DNA, similar to bibenzyl synthase, clone: De324C2	Dendrobium ...	235	235	21%	2e-56	95.30%	1034	LC771167.1
Oncidium Gower Ramsey chalcone synthase (CHS2) gene, complete cds	Oncidium hy...	233	233	24%	6e-56	91.33%	3264	DQ118024.1
Dendrobium officinale bibenzyl synthase mRNA, complete cds	Dendrobium ...	231	231	21%	2e-55	94.67%	1173	MH992134.1
PREDICTED: Dendrobium catenatum bibenzyl synthase (LOC110115249), mRNA	Dendrobium ...	231	231	21%	2e-55	94.67%	1472	XM_020848439.2
Dendrobium kingianum De134 DNA, similar to bibenzyl synthase, clone: De134C6	Dendrobium ...	231	231	21%	2e-55	94.12%	804	LC771172.1
Dendrobium nobile De286 DNA, similar to bibenzyl synthase, clone: De286C12	Dendrobium ...	231	231	20%	2e-55	95.24%	1034	LC771154.1
Dendrobium sinense bibenzyl synthase 1 mRNA, complete cds	Dendrobium ...	231	231	21%	2e-55	93.59%	1470	OP887149.1
Dendrobium fimbriatum De351 DNA, similar to bibenzyl synthase, clone: De351C1	Dendrobium f...	230	230	21%	8e-55	94.63%	1033	LC771160.1
Dendrobium officinale isolate P44 bibenzyl synthase-like protein mRNA, complete cds	Dendrobium ...	230	230	21%	8e-55	94.08%	1173	OR727875.1
Dendrobium amboinense De344 DNA, similar to bibenzyl synthase, clone: De344C26	Dendrobium ...	226	226	21%	1e-53	92.95%	1023	LC771169.1
Dendrobium fimbriatum De351 DNA, similar to bibenzyl synthase, clone: De351C11	Dendrobium f...	226	226	21%	1e-53	92.95%	1029	LC771161.1
Dendrobium officinale isolate TPSH-1 BS1 (BS1) mRNA, complete cds	Dendrobium ...	226	226	21%	1e-53	94.00%	1173	OQ458714.1
Dendrobium officinale bibenzyl synthase 8 mRNA, complete cds	Dendrobium ...	226	226	21%	1e-53	94.00%	1173	OM809168.1
Phalaenopsis sp. 'True Lady' chalcone synthase homolog mRNA, complete cds	Phalaenopsis...	226	226	21%	1e-53	92.95%	1479	U88077.1
Dendrobium speciosum De343 DNA, similar to bibenzyl synthase, clone: De343C11	Dendrobium ...	220	220	21%	5e-52	92.81%	962	LC771170.1
Dendrobium chrysotoxum De268 DNA, similar to bibenzyl synthase, clone: De268C20	Dendrobium ...	220	220	21%	5e-52	92.31%	1028	LC771163.1
PREDICTED: Phalaenopsis equestris bibenzyl synthase-like (LOC110018892), mRNA	Phalaenopsis...	220	220	21%	5e-52	92.31%	1639	XM_020716366.1
Dendrobium nobile isolate R2-P44 bibenzyl synthase-like protein mRNA, complete cds	Dendrobium ...	220	220	21%	5e-52	93.33%	1173	OR687304.1
Phalaenopsis hybrid cultivar isolate CHS_PSY chalcone synthase (chs) gene, partial cds	Phalaenopsis...	220	220	21%	5e-52	92.81%	733	KR184098.1
Phalaenopsis gigantea isolate CHS_PGG chalcone synthase (chs) gene, partial cds	Phalaenopsis...	220	220	21%	5e-52	92.81%	733	KR184097.1
Phalaenopsis celebensis isolate CHS_PCE chalcone synthase (chs) gene, partial cds	Phalaenopsis...	220	220	21%	5e-52	92.81%	733	KR184096.1
Phalaenopsis hybrid cultivar isolate CHS_PSS chalcone synthase (chs) gene, partial cds	Phalaenopsis...	220	220	21%	5e-52	92.81%	733	KR184095.1
Phalaenopsis hybrid cultivar isolate CHS_PPH37 chalcone synthase (chs) gene, partial ...	Phalaenopsis...	220	220	21%	5e-52	92.81%	733	KR184094.1
Phalaenopsis violacea isolate CHS_PVI chalcone synthase (chs) gene, partial cds	Phalaenopsis...	220	220	21%	5e-52	92.81%	733	KR184093.1
Phalaenopsis cornu-cervi isolate CHS_PCC chalcone synthase (chs) gene, partial cds	Phalaenopsis...	220	220	21%	5e-52	92.81%	733	KR184092.1
Phalaenopsis bellina isolate CHS_PBE chalcone synthase (chs) gene, partial cds	Phalaenopsis...	220	220	21%	5e-52	92.81%	733	KR184091.1
Phalaenopsis amabilis isolate CHS_PAB chalcone synthase (chs) gene, partial cds	Phalaenopsis...	220	220	21%	5e-52	92.81%	733	KR184090.1
Phalaenopsis amabilis isolate CHS_PAM chalcone synthase (chs) gene, partial cds	Phalaenopsis...	220	220	21%	5e-52	92.81%	733	KR184089.1
Phalaenopsis hybrid cultivar chalcone synthase pseudogene, complete sequence	Phalaenopsis...	220	220	21%	5e-52	92.81%	1282	AY825503.1
Phalaenopsis hybrid cultivar chalcone synthase pseudogene, complete sequence	Phalaenopsis...	220	220	21%	5e-52	92.81%	1282	AY825503.1
Phalaenopsis x Doritaenopsis hybrid cultivar chalcone synthase gene, complete cds	Phalaenopsis...	220	220	21%	5e-52	92.81%	1499	AY282575.1
Phalaenopsis sp. 'pSPORT1' BibSy811 mRNA for bibenzyl synthase	Phalaenopsis...	220	220	21%	5e-52	92.81%	1525	X79904.1
Phalaenopsis sp. 'pSPORT1' BibSy212 mRNA for bibenzyl synthase	Phalaenopsis...	220	220	21%	5e-52	92.81%	1503	X79903.1
Oncidium hybrid cultivar chalcone synthase (CHS1) gene, complete cds	Oncidium hy...	217	217	22%	7e-51	91.72%	5787	DQ118023.2
Dendrobium chrysotoxum De268 DNA, similar to bibenzyl synthase, clone: De268C21	Dendrobium ...	215	215	21%	2e-50	91.67%	1029	LC771162.1
PREDICTED: Phalaenopsis equestris bibenzyl synthase (LOC110018893), mRNA	Phalaenopsis...	215	215	21%	2e-50	91.67%	1462	XM_020716367.1
Bletilla striata chalcone synthase (CHS) mRNA, complete cds	Bletilla striata	213	213	20%	8e-50	92.62%	1629	KF812890.1
Dendrobium fimbriatum De351 DNA, similar to bibenzyl synthase, clone: De351C2	Dendrobium f...	209	209	21%	1e-48	91.03%	1029	LC771158.1
Dendrobium moschatum De209 DNA, similar to bibenzyl synthase, clone: De209C8	Dendrobium ...	206	206	24%	1e-47	88.44%	1042	LC771166.1
Dendrobium fimbriatum De351 DNA, similar to bibenzyl synthase, clone: De351C12	Dendrobium f...	204	204	21%	5e-47	90.38%	1005	LC771159.1
Oncidium Gower Ramsey chalcone synthase (CHS3) gene, complete cds	Oncidium hy...	187	187	21%	5e-42	89.33%	3637	DQ118022.1
Paphiopedilum concolor chalcone synthase (CHS) gene, partial cds	Paphiopedilu...	121	121	19%	5e-22	82.27%	1307	JQ030898.1
Paphiopedilum purpuratum chalcone synthase (CHS) gene, partial cds	Paphiopedilu...	117	117	19%	7e-21	82.01%	1325	JQ030905.1
Paphiopedilum wenshanense chalcone synthase (CHS) gene, partial cds	Paphiopedilu...	115	115	19%	2e-20	81.56%	1310	JQ030906.1
Paphiopedilum bellatulum chalcone synthase (CHS) gene, partial cds	Paphiopedilu...	115	115	19%	2e-20	81.56%	1307	JQ030897.1
Paphiopedilum concolor chalcone synthase (CHS) mRNA, complete cds	Paphiopedilu...	110	110	19%	1e-18	80.85%	1424	JQ030889.1
Paphiopedilum helenae chalcone synthase (CHS) gene, partial cds	Paphiopedilu...	106	106	19%	1e-17	80.58%	1321	JQ030902.1
Galerucella nymphaeae genome assembly, chromosome: 2	Galerucella n...	56.5	56.5	4%	0.015	100.00%	24227329	OZ021706.1

Figure S3. Homology comparison result of the 12st gene fragment in Table S2.

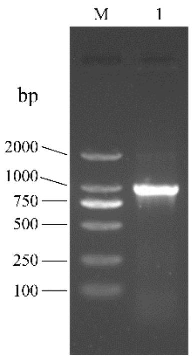

Figure S4. Electrophoresis of full-length PCR product of DoBS1 gene cloning. M: DNA marker (100-2000 bp)；Lane 1：DoBS1 gene

CATATGCCGAGCCTGGAAAGTATTAAGAAAGCCCCGCGCGCAGATGGCTTTGCAAGCATTCTGGCCATTGG
CCGTGCAAATCCGGAAAATTTTATTGAACAGAGTGCATATCCGGATCTGTTTTTCCGCATTACCAATAGCGAA
CATCTGGTGGATCTGAAAAATAAGTTTAAACGTATCTGCGATAAGACCGCAATTCGTAAACGTCATTTTGTGT
GGACCGAAGAGTTTATTACCGCAAATCCGTGTTTTAGTACCTTTATGGATAAAAGCCTGAATATTCGCCAGGA
AGTGGCCATTCGCGAAATTCCGAAACTGGGCGCAGAAGCCGCAACCAAAGCAATTCAGGAATGGGGCCAG
CCGAAAAGTCGCATTACCCATCTGATTTTCTGTACCACCAGTGGTATGGATCTGCCGGGCGCAGATTATCAGC
TGACCCAGATTCTGGGTCTGAATCCGAATGTGGAACGTGTGATGCTGTATCAGCAGGGTTGTTTTGCAGGTG
GTACAACCATTCGCCTGGCCAAATGTCTGGCCGAAAGCCGTAAAGGCGCCCGTGTGCTGGTTGTGTGCGCA
GAAACCACCACCGTTCTGTTTCGCGGTCCGAGTGAAGAACATCAGGATGATCTGGTTACCCAGGCACTGTTT
GCCGATGGCGCCAGTGCACTGATTGTGGGCGCAGATCCGGATGAAGCAGCCCATGAACGTGCAAGTTTTGT
TATTGTTAGCACCAGCCAGGTGCTGCTGCCGGATAGTGCAGGTGCCATTGGCGGTCATGTGAGTGAAGGCG
GCCTGCTGGCCACCCTGCATCGTGATGTTCCGCATATTGTTAGTAAAAATGTTGAAAAGTGCCTGGAAGAAG
CATTCACTCCGTTTGGTATTAGCGATTGGAATAGCATTTTCTGGGTTCCGCATCCGGGCGGCCGTGCCATTCT
GGATCAGGTGGAAGAACGTGTTGGTCTGAAACCGGAAAAACTGCTGGTGAGTCGCCATGTGCTGGCCGAA
TATGGCAATATGAGTAGCGTGTGTGTTCATTTTGCACTGGATGAAATGCGTAAAACCAGTGCCCGCGAAGGT
AAAGCAACCACCGGCGAAGGTCTGGAATGGGGCGTTCTGTTTGGCTTTGGCCCGGGCCTGACCGTTGAAA
CCGTTGTGCTGCGCAGTGTGCCGCTGTAACTCGAG

Figure S5. Results of DoBS1 gene sequencing.

1	ATGCCGAGCCTGGAAAGTATTAAGAAAGCCCCGCGCGCAGATGGCTTTGCAAGCATTCTG
1	**M P S L E S I K K A P R A D G F A S I L**
61	GCCATTGGCCGTGCAAATCCGGAAAATTTTATTGAACAGAGTGCATATCCGGATCTGTTT
21	**A I G R A N P E N F I E Q S A Y P D L F**
121	TTCCGCATTACCAATAGCGAACATCTGGTGGATCTGAAAAATAAGTTTAAACGTATCTGC
41	**F R I T N S E H L V D L K N K F K R I C**
181	GATAAGACCGCAATTCGTAAACGTCATTTTGTGTGGACCGAAGAGTTTATTACCGCAAAT
61	**D K T A I R K R H F V W T E E F I T A N**
241	CCGTGTTTTAGTACCTTTATGGATAAAAGCCTGAATATTCGCCAGGAAGTGGCCATTCGC
81	**P C F S T F M D K S L N I R Q E V A I R**
301	GAAATTCCGAAACTGGGCGCAGAAGCCGCAACCAAAGCAATTCAGGAATGGGGCCAGCCG
101	**E I P K L G A E A A T K A I Q E W G Q P**
361	AAAAGTCGCATTACCCATCTGATTTTCTGTACCACCAGTGGTATGGATCTGCCGGGCGCA
121	**K S R I T H L I F C T T S G M D L P G A**
421	GATTATCAGCTGACCCAGATTCTGGGTCTGAATCCGAATGTGGAACGTGTGATGCTGTAT
141	**D Y Q L T Q I L G L N P N V E R V M L Y**
481	CAGCAGGGTTGTTTTGCAGGTGGTACAACCATTCGCCTGGCCAAATGTCTGGCCGAAAGC
161	**Q Q G C F A G G T T I R L A K C L A E S**
541	CGTAAAGGCGCCCGTGTGCTGGTTGTGTGCGCAGAAACCACCACCGTTCTGTTTCGCGGT
181	**R K G A R V L V V C A E T T T V L F R G**
601	CCGAGTGAAGAACATCAGGATGATCTGGTTACCCAGGCACTGTTTGCCGATGGCGCCAGT
201	**P S E E H Q D D L V T Q A L F A D G A S**
661	GCACTGATTGTGGGCGCAGATCCGGATGAAGCAGCCCATGAACGTGCAAGTTTTGTTATT
221	**A L I V G A D P D E A A H E R A S F V I**
721	GTTAGCACCAGCCAGGTGCTGCTGCCGGATAGTGCAGGTGCCATTGGCGGTCATGTGAGT
241	**V S T S Q V L L P D S A G A I G G H V S**
781	GAAGGCGGCCTGCTGGCCACCCTGCATCGTGATGTTCCGCATATTGTTAGTAAAAATGTT
261	**E G G L L A T L H R D V P H I V S K N V**
841	GAAAAGTGCCTGGAAGAAGCATTCACTCCGTTTGGTATTAGCGATTGGAATAGCATTTTC
281	**E K C L E E A F T P F G I S D W N S I F**
901	TGGGTTCCGCATCCGGGCGGCCGTGCCATTCTGGATCAGGTGGAAGAACGTGTTGGTCTG
301	**W V P H P G G R A I L D Q V E E R V G L**
961	AAACCGGAAAAACTGCTGGTGAGTCGCCATGTGCTGGCCGAATATGGCAATATGAGTAGC
321	**K P E K L L V S R H V L A E Y G N M S S**
1021	GTGTGTGTTCATTTTGCACTGGATGAAATGCGTAAAACCAGTGCCCGCGAAGGTAAAGCA
341	**V C V H F A L D E M R K T S A R E G K A**
1081	ACCACCGGCGAAGGTCTGGAATGGGGCGTTCTGTTTGGCTTTGGCCCGGGCCTGACCGTT
361	**T T G E G L E W G V L F G F G P G L T V**
1141	GAAACCGTTGTGCTGCGCAGTGTGCCGCTGTAA
381	**E T V V L R S V P L ***

Figure S6. The DoBS1 gene coding sequence and amino acids. Bold-type letter: Amino acids; No bold: Nucleotide; *: Termination codon.

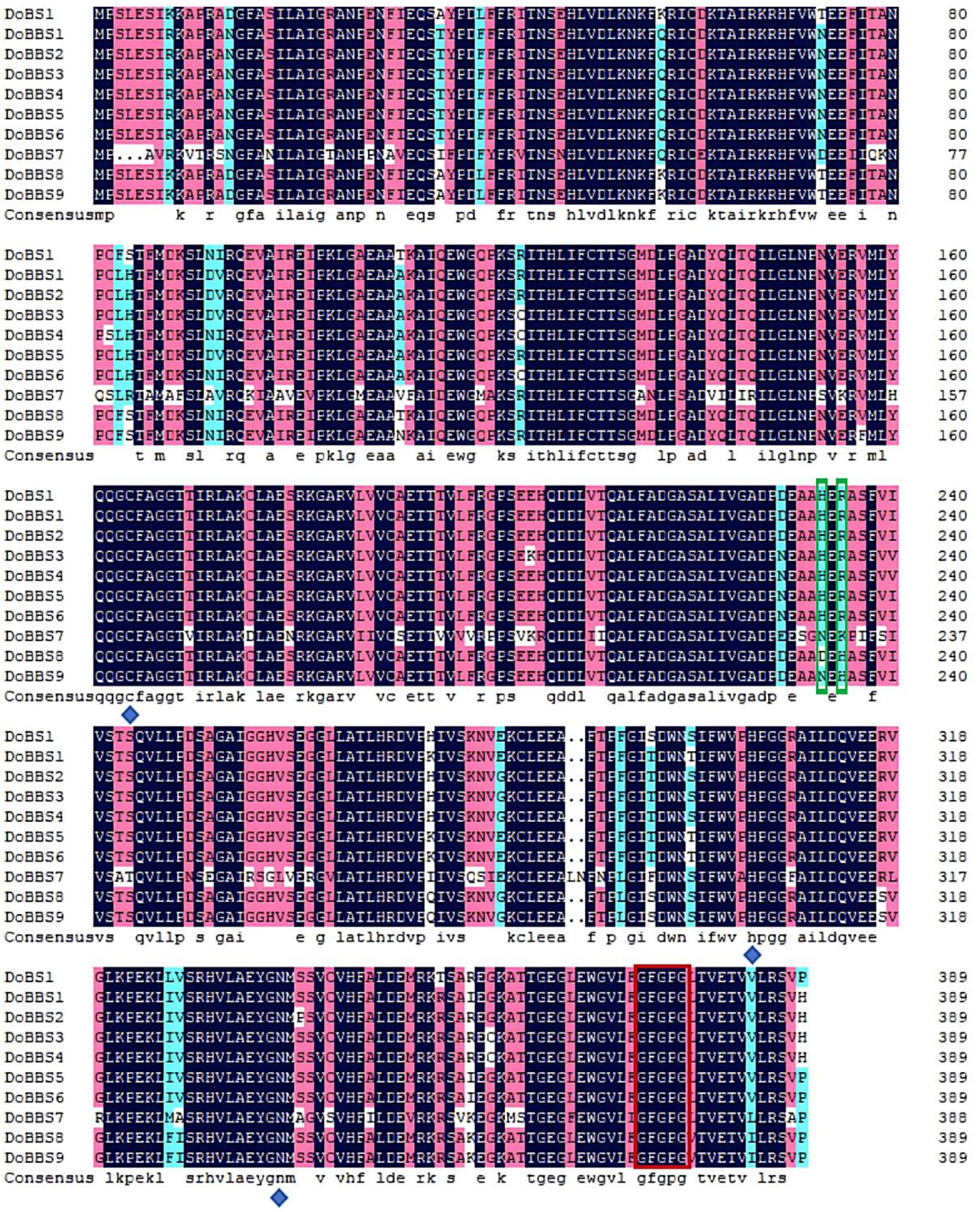

Figure S7. Comparison of amino acid sequences of BBS derived from the same plant (*D.officinale*). Multiple sequence alignment was calculated with the DNAMAN package. Black shading indicates the homology of amino acids, while red and blue shading indicates amino acids with different similarity. The conserved catalytic residues in plant type III PKS (Cys164, His304 and Asn337, DoBS1 numbers) are represented by diamonds. Additionally, the highly conserved sequence G373FGPG (DoBS1 number) is indicated by red boxes.

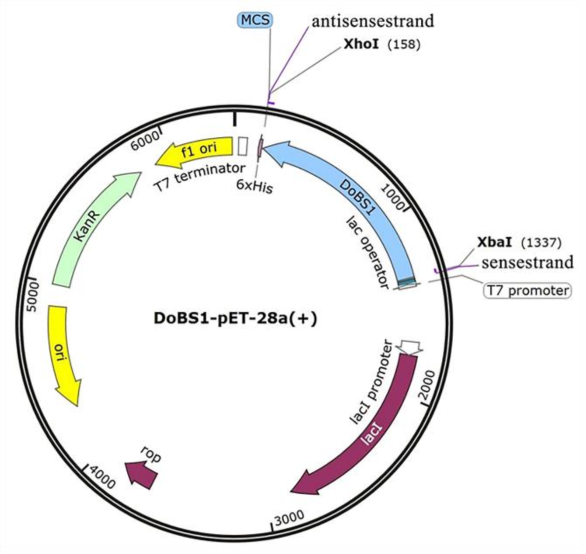

Figure S8. Construction of DoBS1 into PET-28a vector. Image of the integration of the target gene into the PET-28a vector in result 2.4.

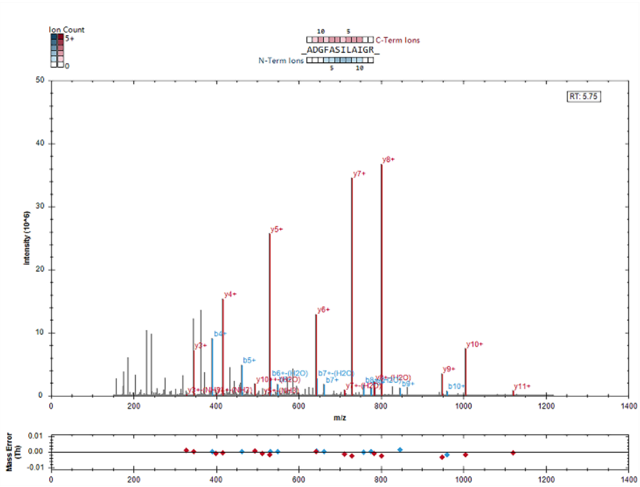

Figure S9. MS /MS analysis of peptide ion 3 (ADGFASILAIGR).

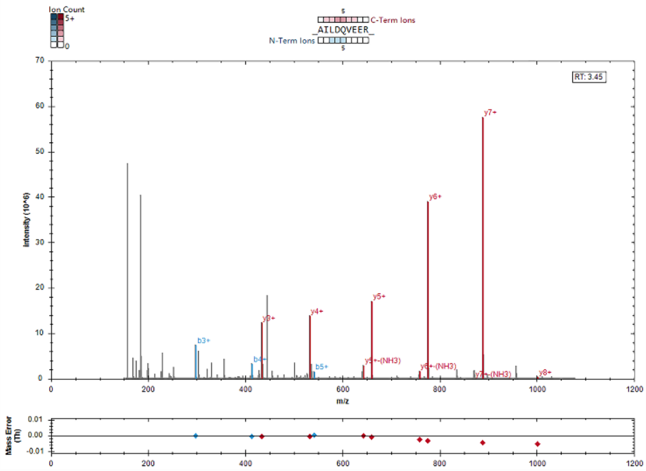

Figure S10. MS /MS analysis of peptide ion 4 (AILDQVEER).

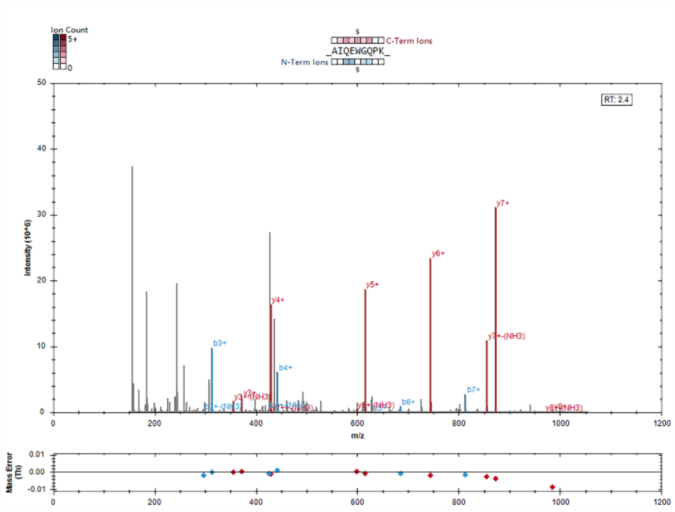

Figure S11. MS /MS analysis of peptide ion 5 (AIQEWGQPK).

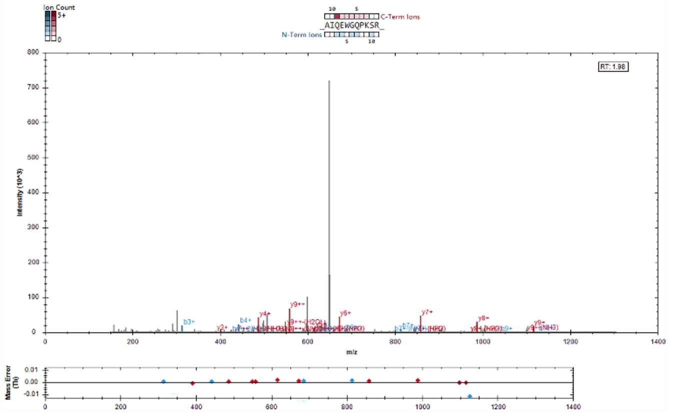

Figure S12. MS /MS analysis of peptide ion 6 (AIQEWGQPKSR).

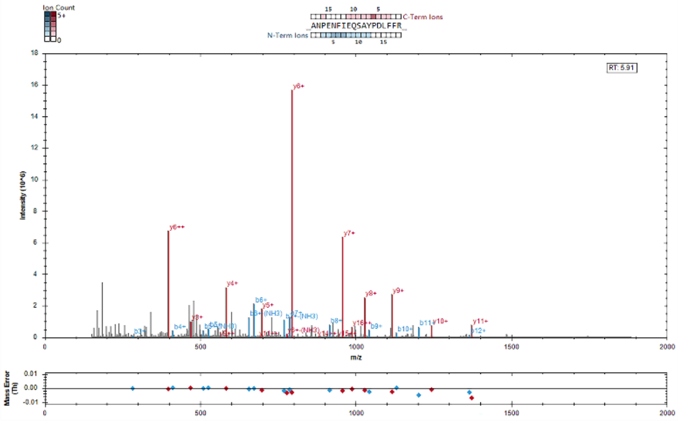

Figure S13. MS /MS analysis of peptide ion 7 (ANPENFIEQSAYPDLFFR).

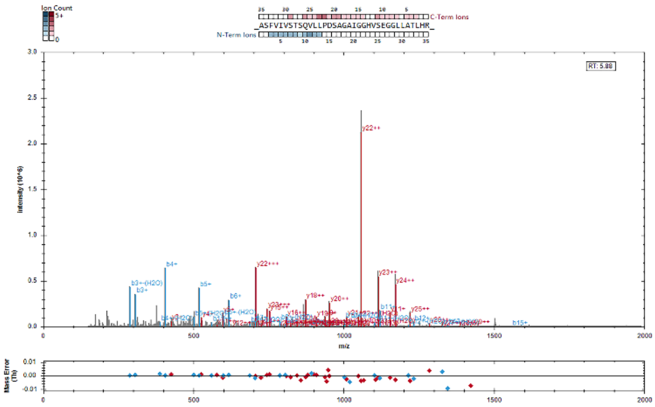

Figure S14. MS /MS analysis of peptide ion 8 (ASFVIVSTSQVLLPDSAGAIGGHVSEGGLLATLHR).

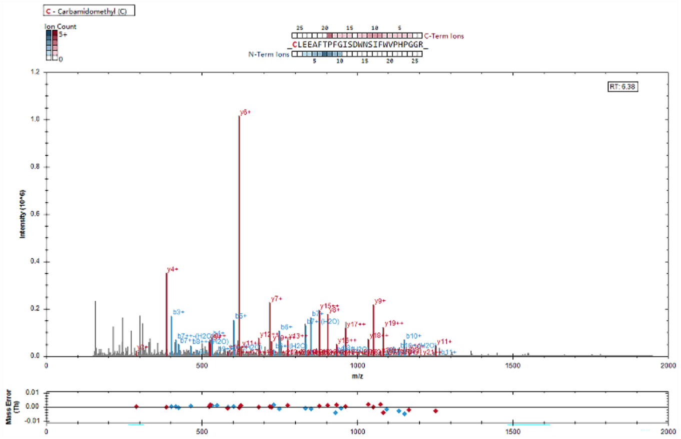

Figure S15. MS /MS analysis of peptide ion 9 (CLEEAFTPFGISDWWSIFWVPHPGGR).

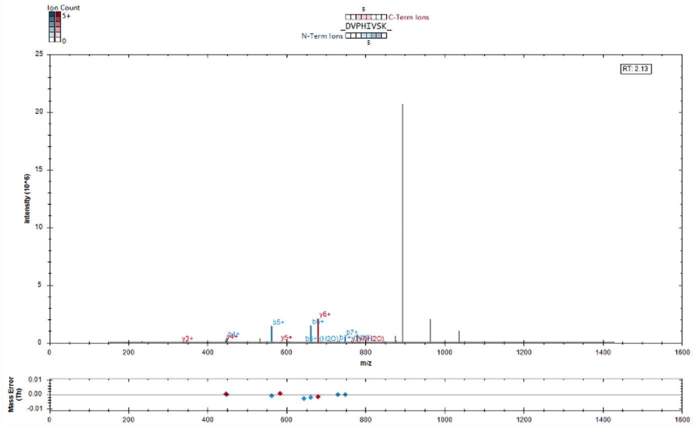

Figure S16. MS /MS analysis of peptide ion 10 (DVPHIVSK).

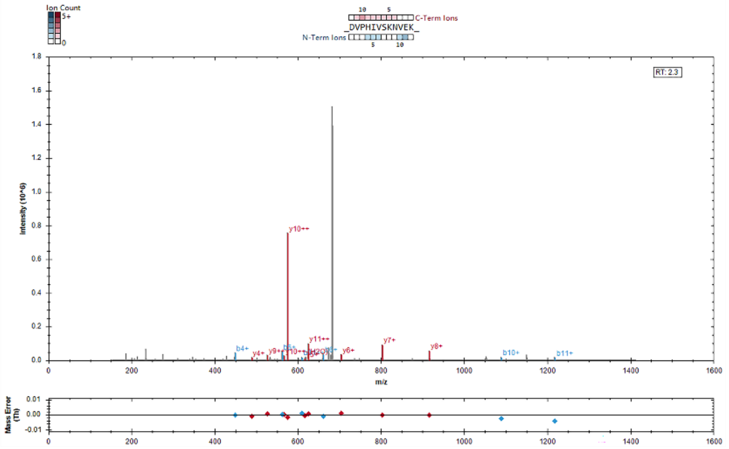

Figure S17. MS /MS analysis of peptide ion 11 (DVPHIVSKNVEK).

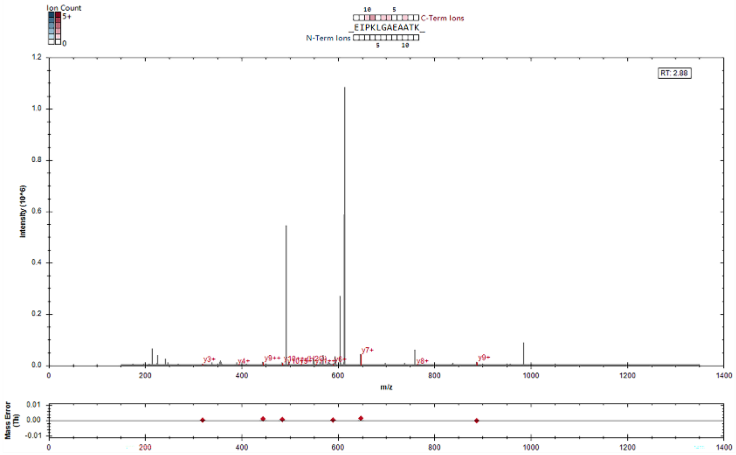

Figure S18. MS /MS analysis of peptide ion 12 (EIPKLGAEAATK).

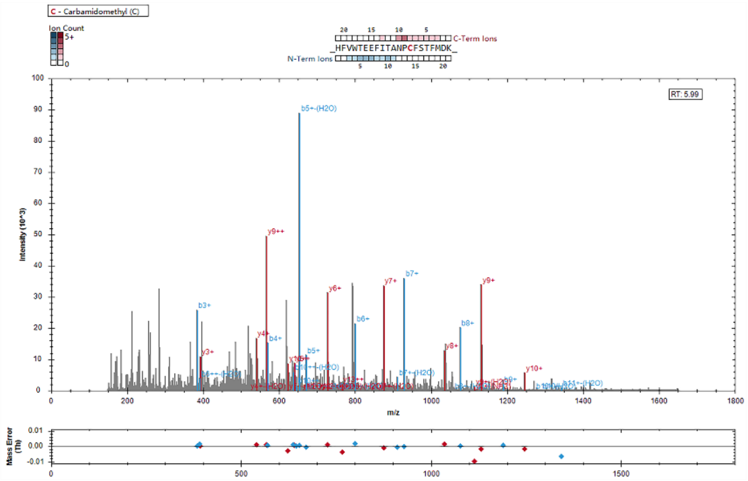

Figure S19 MS /MS analysis of peptide ion 13 (HFVWTEEFITANPCFSTFMDK).

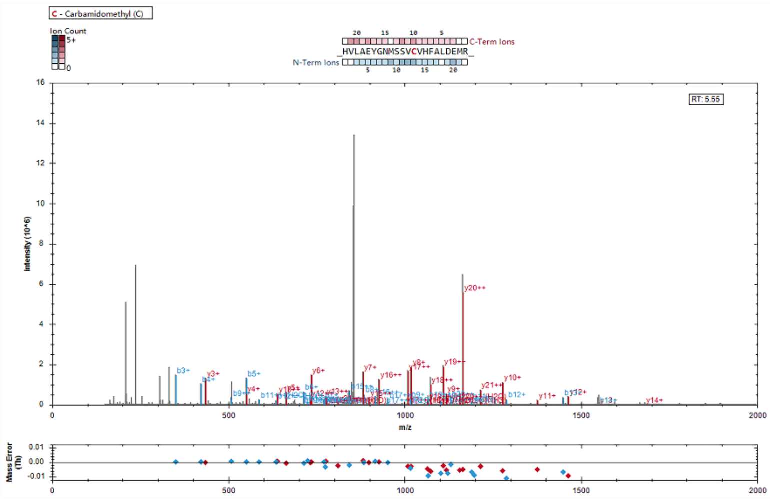

Figure S20. MS /MS analysis of peptide ion 14 (HVLAEYGNMSSVCVHFALDEMR).

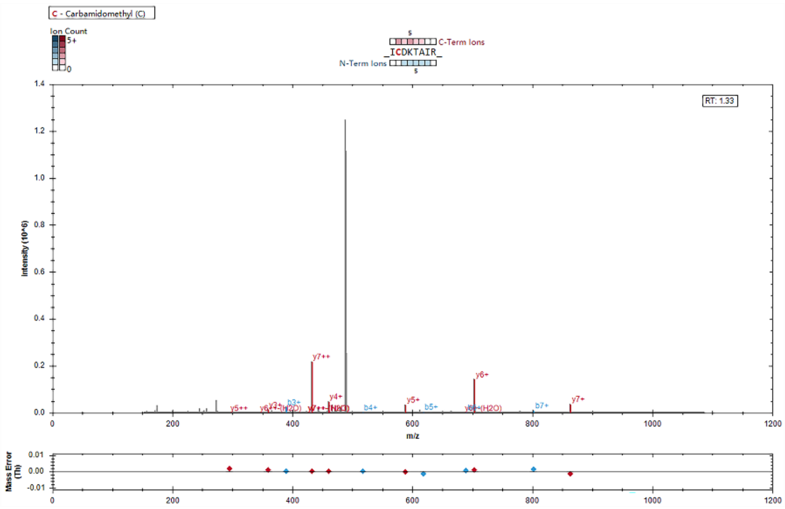

Figure S21. MS /MS analysis of peptide ion 15 (ICDKTAIR).

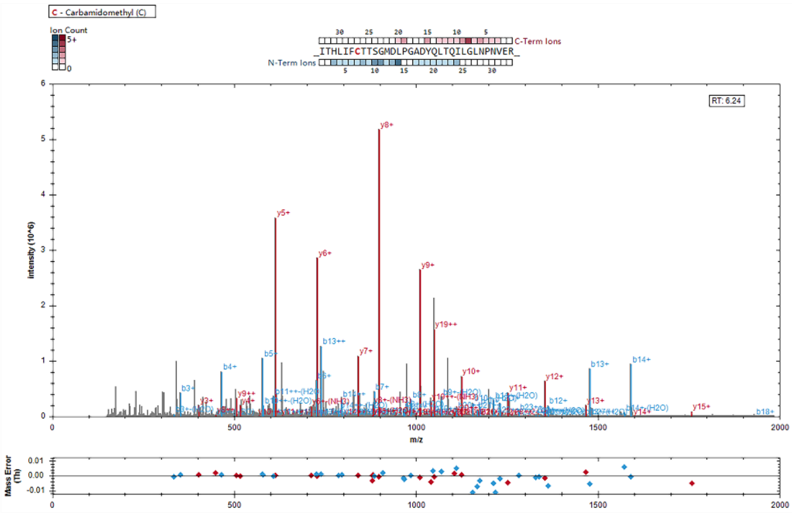

Figure S22. MS /MS analysis of peptide ion 16 (ITHLIFCTTSGMDLPGADYQLTQILGLNPNVER).

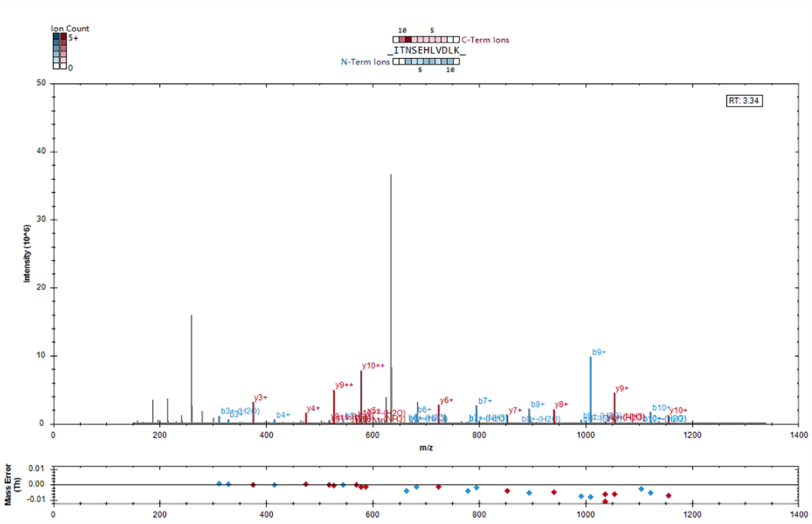

Figure S23. MS /MS analysis of peptide ion 17 (ITNSEHLVDLK).

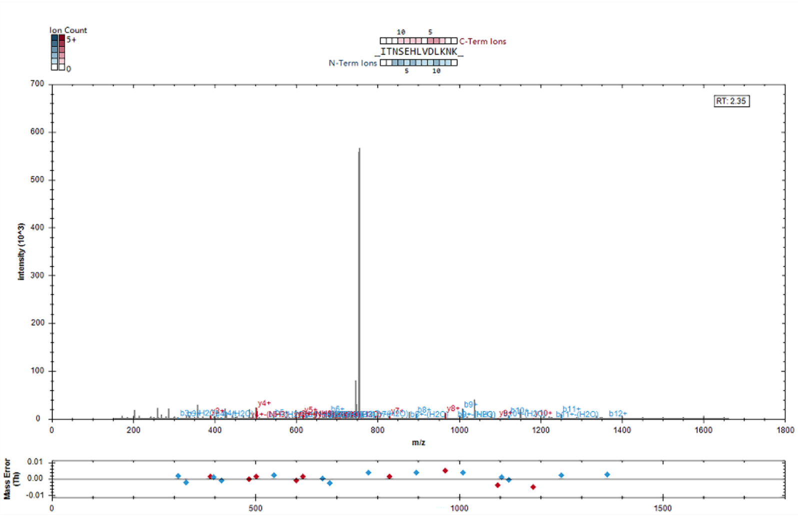

Figure S24. MS /MS analysis of peptide ion 18 (ITNSEHLVDLKNK).

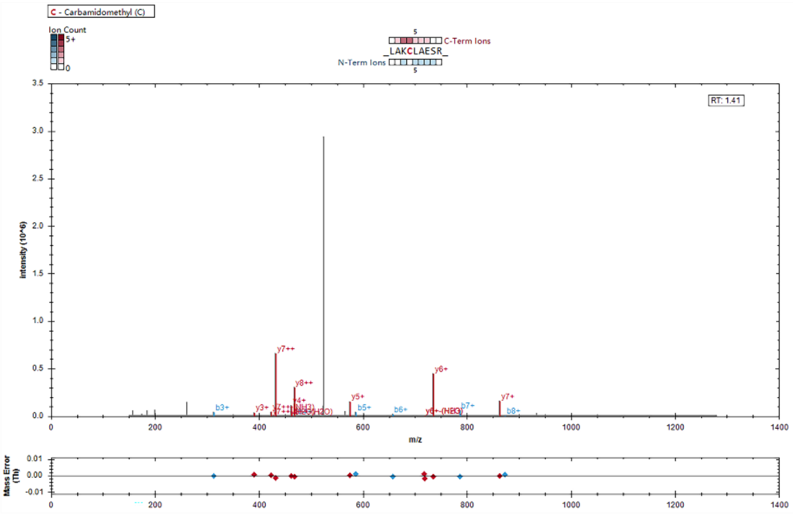

Figure S25. MS /MS analysis of peptide ion 19 (LAKCLAESR).

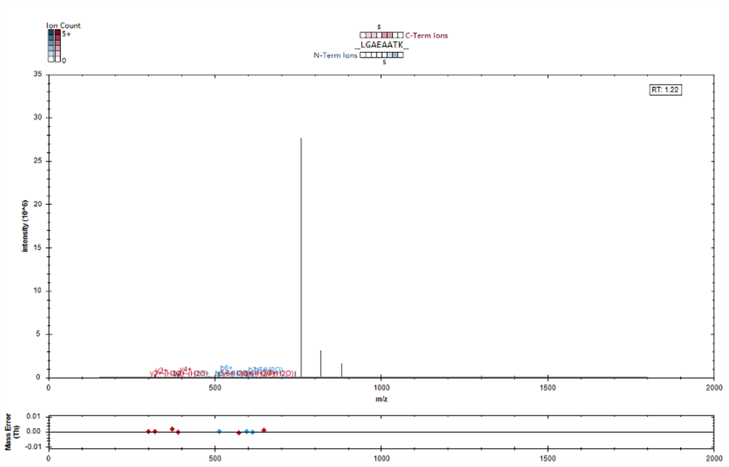

Figure S26. MS /MS analysis of peptide ion 20 (LGAEAATK).

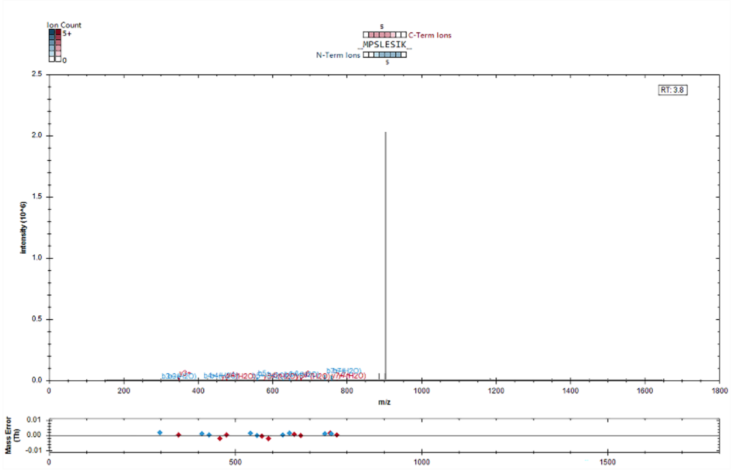

Figure S27. MS /MS analysis of peptide ion 21 (MPSLESIK).

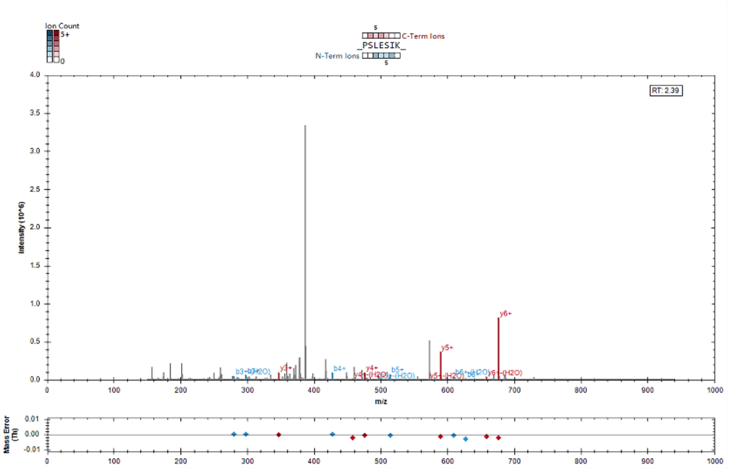

Figure S28. MS /MS analysis of peptide ion 22 (PSLESIK).

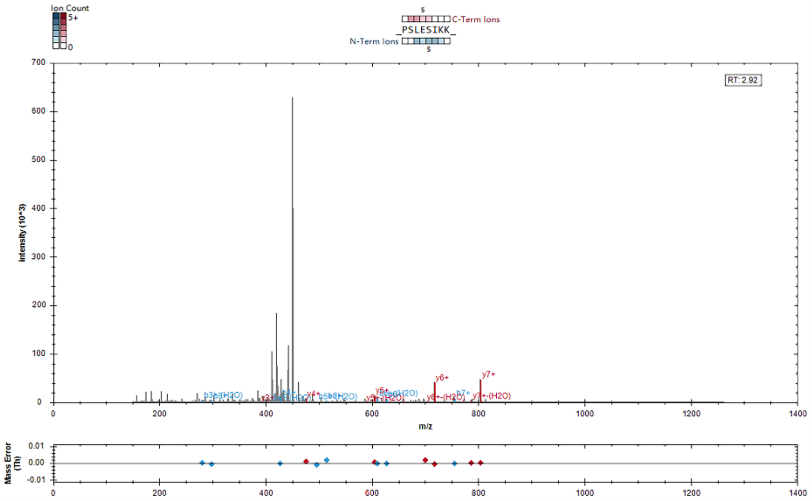

Figure S29. MS /MS analysis of peptide ion 23 (PSLESIKK).

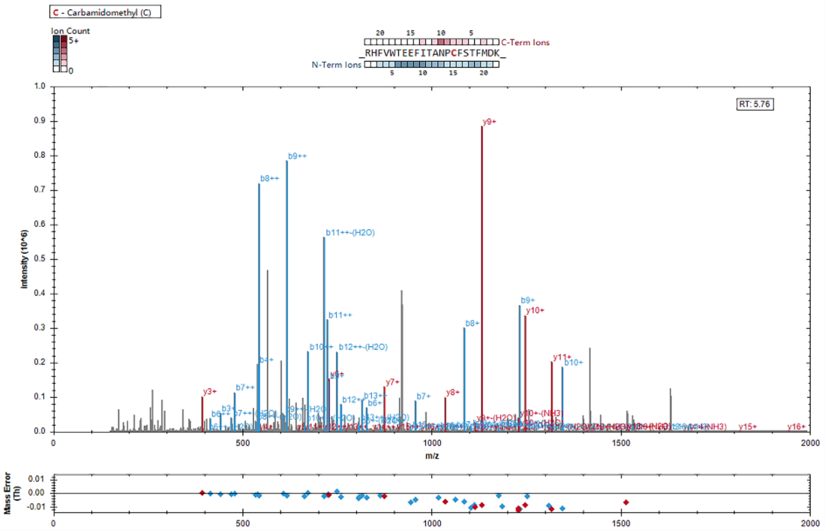

Figure S30. MS /MS analysis of peptide ion 24 (RHFVWTEEFITANPCFSTFMDK).

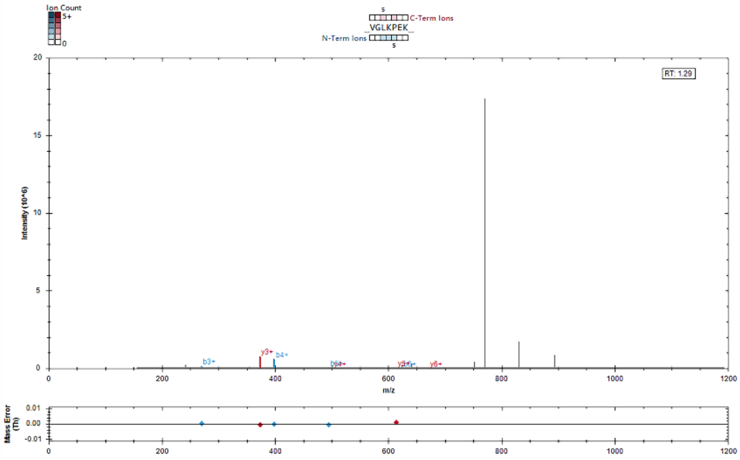

Figure S31. MS /MS analysis of peptide ion 25 (VGLKPEK).

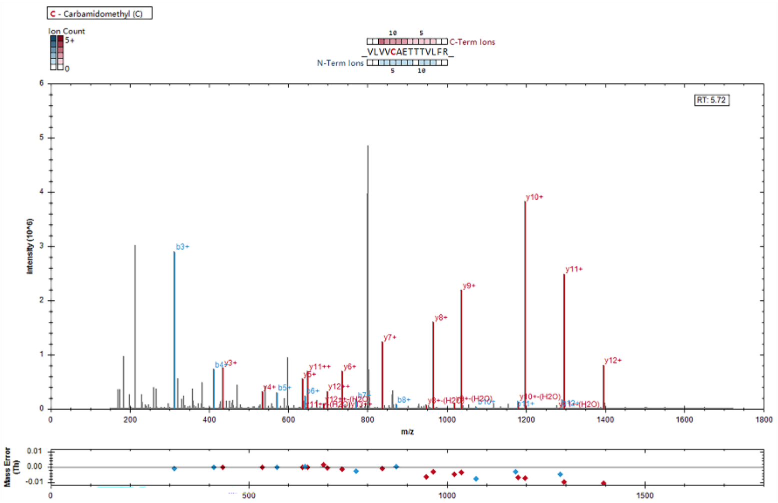

Figure S32. MS /MS analysis of peptide ion 26 (VLVVCAETTTVLFR).

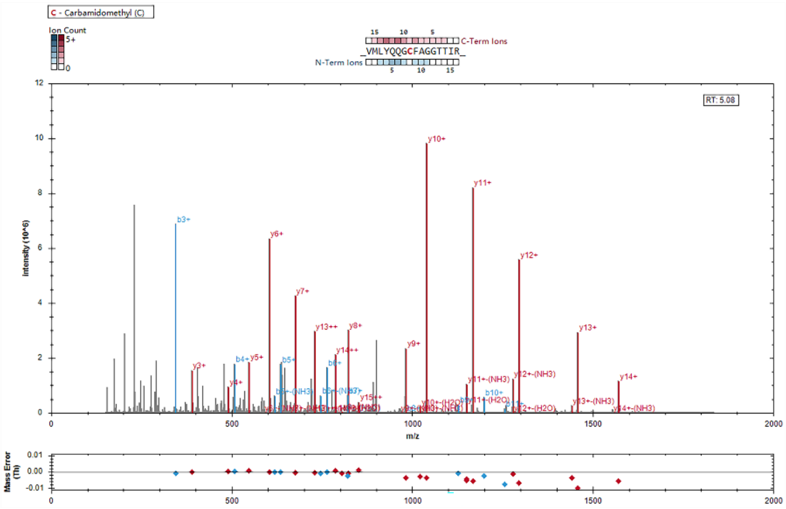

Figure S33. MS /MS analysis of peptide ion 27 (VMLYQQGCFAGGTTIR).

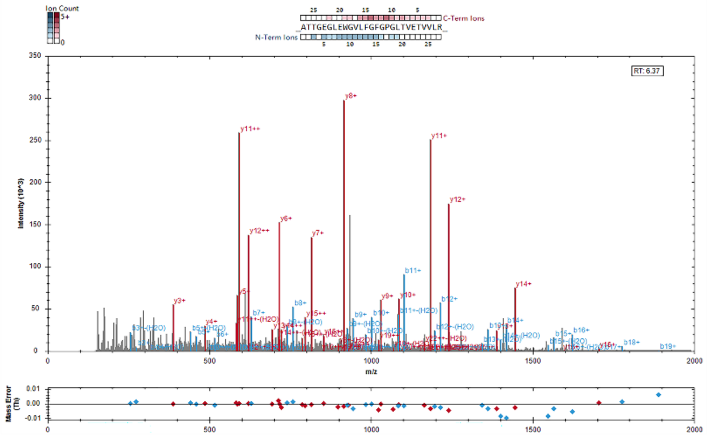

Figure S34. MS /MS analysis of peptide ion 28 (ATTGEGLEWGVLFGFGPGLTVETVVLR).

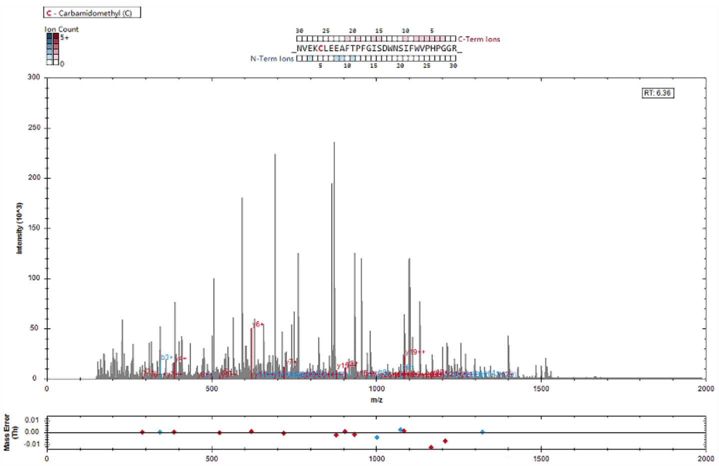

Figure S35. MS /MS analysis of peptide ion 29 (NVEKCLEEAFTPFGISDWWSIFWVPHPGGR).

Mass spectra of the remaining peptide fragments in Result 2.5.

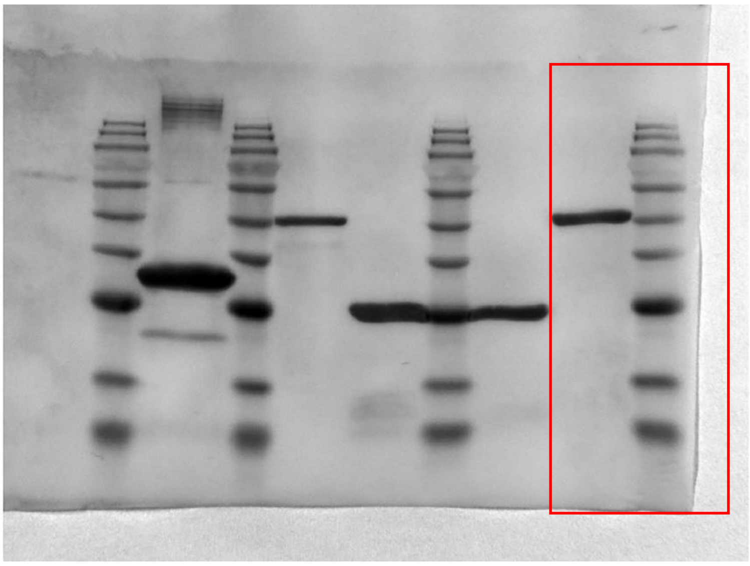

Figure S36. Original Western Blot image. The box indicate lanes shown in Figure 9 (a) of the Manuscript. Original picture of the Western blot in Figure 7(a) in Results 2.5.

Supplementary Material

Construct phylogenetic tree source files

Pseudomonas fluorescens PhlD(AAB48106)

>AAB48106.1 PhlD [Pseudomonas fluorescens Q2-87]

MSTLCKPSLLFPHYKITQQQMIDHLEQLHDDHPRMALAKRMIQNTQVNERYLVLPIDELAVHTGFTHRSI
VYEREARRMSSIAARQAIENAGLTTDDIRMVAVTSCTGFMMPSLTAHLINDLGLRTSTVQLPIAQLGCVA
GAAAINRANDFASLSPDNHALIVSLEFSSLCYQPQDTKLHAFISAALFGDAVSACVMRADDKAPGFKIAK
TGSYFLPDSEHYIKYDVKDSGFHFTLDKAVMNSIKDVAPMMEELNFETFNQHCAQNDFFIFHTGGRKILD
ELVLQLDLEPGRVAQSRDSLSEAGNIASVVVFDVLKRQFDSGPANGATGMLAAFGPGFTAEMAVGKWVA

Streptomyces griseus RppA (BAA33495)

>BAA33495.1 orf-1 [Streptomyces griseus]

MATLCRPAIAVPEHVITMQQTLDLARETHAGHPQRDLVLRLIQNTGVQTRHLVQPIEKTLAHPGFEVRNQ
VYEAEAKTRVPEVVRRALANAETEPSEIDLIVYVSCTGFMMPSLTAWIINSMGFRPETRQLPIAQLGCAA
GGAAINRAHDFCVAYPDSNVLIVSCEFCSLCYQPTDIGVGSLLSNGLFGDALSAAVVRGQGGTGMRLERN
GSHLVPDTEDWISYAVRDTGFHFQLDKRVPGTMEMLAPVLLDLVDLHGWSVPNMDFFIVHAGGPRILDDL
CHFLDLPPEMFRYSRATLTERGNIASSVVFDALARLFDDGGAAESAQGLIAGFGPGITAEVAVGSWAKEG
LGADVGRDLDELELTAGVALSG

Mycobacterium tuberculosis PKS18 (A70958)替代为（PKS18_MYCTO）

>sp|P9WPF0.1|PKS18_MYCTO RecName: Full=Alpha-pyrone synthesis polyketide synthase-like Pks18; AltName: Full=Alpha-pyrone synthesis polyketide synthase type III Pks18; AltName: Full=Chalcone synthase-like protein; Short=CHS-like

MNVSAESGAPRRAGQRHEVGLAQLPPAPPTTVAVIEGLATGTPRRVVNQSDAADRVAELFLDPGQRERIP
RVYQKSRITTRRMAVDPLDAKFDVFRREPATIRDRMHLFYEHAVPLAVDVSKRALAGLPYRAAEIGLLVL
ATSTGFIAPGVDVAIVKELGLSPSISRVVVNFMGCAAAMNALGTATNYVRAHPAMKALVVCIELCSVNAV
FADDINDVVIHSLFGDGCAALVIGASQVQEKLEPGKVVVRSSFSQLLDNTEDGIVLGVNHNGITCELSEN
LPGYIFSGVAPVVTEMLWDNGLQISDIDLWAIHPGGPKIIEQSVRSLGISAELAAQSWDVLARFGNMLSV
SLIFVLETMVQQAESAKAISTGVAFAFGPGVTVEGMLFDIIRR

Amycolatopsis mediterranei DpgA (CAC48378）

>CAC48378.1 dihydroxyphenylacetic acid synthase [Amycolatopsis balhimycina DSM 5908]

MGVDVSMTTSIEPAEDLSVLSGLTEITRFAGVGTAVSASSYSQSEVLDILDVEDPKIRSVFLNSAIDRRF
LTLPPESPGGGRVSEPQGDLLDKHKELAVDMGCRALEACLKSAGATLSDLRHLCCVTSTGFLTPGLSALI
IRELGIDPHCSRSDIVGMGCNAGLNALNVVAGWSAAHPGELGVVLCSEACSAAYALDGTMRTAVVNSLFG
DGSAALAVISGDGRVPGPRVLKFASYIITDALDAMRYDWDRDQDRFSFFLDPQIPYVVGAHAEIVADRLL
SGTGLRRSDIGHWLVHSGGKKVIDSVVVNLGLSRHDVRHTTGVLRDYGNLSSGSFLFSYERLAEEGVTRP
GDYGVLMTMGPGSTIEMALIQW

Dendrobium officinale DoBS1（WDI23477）

>WDI23477.1 BS1 [Dendrobium officinale]

MPSLESIKKAPRADGFASILAIGRANPENFIEQSAYPDLFFRITNSEHLVDLKNKFKRICDKTAIRKRHF
VWTEEFITANPCFSTFMDKSLNIRQEVAIREIPKLGAEAATKAIQEWGQPKSRITHLIFCTTSGMDLPGA
DYQLTQILGLNPNVERVMLYQQGCFAGGTTIRLAKCLAESRKGARVLVVCAETTTVLFRGPSEEHQDDLV
TQALFADGASALIVGADPDEAAHERASFVIVSTSQVLLPDSAGAIGGHVSEGGLLATLHRDVPHIVSKNV
EKCLEEAFTPFGISDWNSIFWVPHPGGRAILDQVEERVGLKPEKLLVSRHVLAEYGNMSSVCVHFALDEM
RKTSAREGKATTGEGLEWGVLFGFGPGLTVETVVLRSVPL*

Dendrobium catenatum DcBBS（XP 020704098.1）
>XP_020704098.1 bibenzyl synthase [Dendrobium catenatum]
MPSLESIKKAPRADGFASILAIGRANPENFIEQSAYPDLFFRITNSEHLVDLKNKFKRICDKTAIRKRHF
VWTEEFITANPCFSTFMDKSLNIRQEVAIREIPKLGAEAATKAIQEWGQPKSRITHLIFCTTSGMDLPGA
DYQLTQILGLNPNVERVMLYQQGCFAGGTTIRLAKCLAESRKGARVLVVCAETTTVLFRGPSEEHQDDLV
TQALFADGASALIVGADPDEAADEHASFVIVSTSQVLLPDSAGAIGGHVSEGGLLATLHRDVPQIVSKNV
GKCLEEAFTPLGISDWNSIFWVPHPGGRAILDQVEESVGLKPEKLFISRHVLAEYGNMSSVCVHFALDEM
RKRSAKEGKATTGEGLEWGVLFGFGPGVTVETVILRSVPI
Dendrobium officinale bibenzyl synthase-like（WOK44117.1）
>WOK44117.1 bibenzyl synthase-like protein [Dendrobium officinale]
MPSLESIRKAPRANGFASILAIGRANPENFIEQSTYPDFFFRITNSEHLVDLKKKFQRICDKTAIRKRHF
VWNEEFITANPCLHTFMDKSLNIRQEVAIREIPKLGAEAAAKAIQEWGQPKSCITHLIFCTTSGMDLPGA
DYQLTQLLGLNPNIERVMLYQQGCFAGGTTLRLAKCLAESRKGARVLVVCAETTTVLFRGPSEEHQDDLV
TQALFADGASALIVGADPNEAAHERASFVIVSTSQVLLPDSAGAIGGHVSEGGLLATLHRDVPKIVSKNV
EKCLEEAFTPFGITDWNSIFWVPHPGGRAILDQVEERVGLKPEKLLVSRHVLAEYGNMSSVCVHFALDEM
RKRSAREGKATTGEGLEWGVLFGFGPGLTVETVVLRSVPL
Dendrobium sinense bibenzyl synthase1（WHE45950.1）
>WHE45950.1 bibenzyl synthase 1 [Dendrobium sinense]
MPSLESIKKAPRADGFASILAIGRANPENFIEQSAFPDLFFRITNSEHLVDLKNKFKRICDKTAIRKRHF
VWTEEFITANPCFSTFMGKSLNIRQEVAIREIPKLGAEAAAKAIQEWGQPKSRITHLIFCTTSGMDLPGA
DYQLTQILGLNPNVERVMLYQQGCFAGGTTLRLAKCLAESRKGARVLVVCAETTTVLFRAPSHEHQDDLV
TQALFADGASALIVGADPDEAADEHASFVIVSTSQVLLPESAGAIGGHVSEGGLLATLHRDVPQIVSKNV
GKCLEEAFTPLGISDWNSIFWVPHPGGRAILDQVEERVGLKPEKLFISRHVLAEYGNMSSVCVHFALDEM
RKRSAKEGKATTGEGLEWGVLFGFGPGLTVETVVLRSVPI
Dendrobium catenatum Bibenzyl synthase-like（ XP 020690099.2）
>XP_020690099.2 bibenzyl synthase-like [Dendrobium catenatum]
MPSLESIRKAPRANGFASILAIGRANPENFIEQSTYPDFFFRITNSEHLVDLKKKFQRICDKTAIRKRHF
VWNEEFITTNPCLHTFMDKSLDVRQEVAIREIPKLGAEAAATAIQEWGQPKSRITHLIFCTTSGMDLPGA
DYQLTQILGLNPNVERVMLYQQGCFAGGTTLRLAKCLAESRKGARVLVVCAETTTVLFRGPSEEHQEDLV
TQALFADGASALIVGADPDEAAHERASFVIVSTSQVLLPDSAGAIGGHVSEGGLLATLHRDVPKIVSKNV
EKCLEEAFTPFGITDWNSIFWVPHPGGRAILDLVEERVGLKPEKLLVSRHVLAEYGNMSSVCVHFALDEM
RKRSAIEGKATTGEGLEWGVVFGFGPGLTVETVVLRSVPL
Phalaenopsis equestris bibenzyl synthase（XP_020572026）
>XP_020572026.1 bibenzyl synthase [Phalaenopsis equestris]
MPSLDSIKKAPRADGFASILAIGRANPDNIIEQSAYPDFYFRVTNSEHLVDLKKKFQRICEKTAIRKRHF
VWNEEFLTSNPCFSTFMDKSLNVRQEVAIREIPKLGAKAATKAIEDWGQPKSRITHLIFCTTSGMDLPGA
DYQLTQILGLNPNVERVMLYQQGCFAGGTTLRLAKCLAESRKGARVLVVCAETTTVLFRAPSEEHQDDLV
TQALFADGASAVIVGADPDEAADERASFVIVSTSQVLLPDSAGAIGGHVSEGGLLATLHRDVPQIVSKNV
GKCLEEAFTPFGISDWNSIFWVPHPGGRAILDQVEERVGLKPEKLSVSRHVLAEYGNMSSVCVHFALDEM
RKRSANEGKATTGEGLEWGVLFGFGPGLTVETVVLRSVPL
Dendrobium sinense bibenzyl synthase2（WHE45951.1）
>WHE45951.1 bibenzyl synthase 2 [Dendrobium sinense]
MPSLESIRKAPRANGFASILAIGRANPENFIEQSTYPDFFFRITNSEHLVDLKKKFQRICDKTEIRKRHF
VWNEELITANPCLHTFMDKSLDVRQEFAIREIPKLGAEAAAKAIQEWGQPKSQITHLIFCTTSGMDLPGA

DYQLTQILGLNPNVERVMLYQQGCFAGGTTLRLAKCLAESRKGARVLVVCAETTAVLFRGPSEEHQDDLV
TQALFADGASALIVGADPDEAAHERASFVIVSTSQVLLPDSAGAIGGHVSEGGLLATLHRDVPHIVSKNV
GKCLEEAFTPFGISDWNSIFWVPHPGGRAILDQVEERVGLKPEKLLVSRQVLAEYGNMSSVCVHFALDEM
RKRSAREGKATTGEGLEWGVLFGFGPGVTVETVVLRSVPF

Phalaenopsis hybrid cultivar chalcone synthase（AAV70116）

>AAV70116.1 chalcone synthase [Phalaenopsis hybrid cultivar]
MPSLESIKKAPRADGFASILAIGRANPDNIIEQSAYPDFYFRVTNSEHLVDLKKKFQRICEKTAIRKRHF
VWNEEFLTANPCFSTFMDKSLNVRQEVAISEIPKLGAKAATKAIEDWGQPKSRITHLIFCTTSGMALPGA
DYQLTQILGLNPNVERVMLYQQGCFAGGTTLRLAKCLAESRKGARVLVVCAETTTVLFRAPSEEHQDDLV
TQALFADGASAVIVGADPDEAADERASFVIVSTSQVLLPDSAGAIGGHVSEGGLLATLHRDVPQIVSKNV
GKCLEEAFTPFGISDWNSIFWVPHPGGRAILDQVEERVGLKPEKLSVSRHVLAEYGNMSSVCVHFALDEM
RKRSANEGKATTGEGLEWGVLFGFGPGLTVETVVLRSVPL

Dendrobium catenatum Bibenzyl synthase-like（XP 020690097）

>XP_020690097.1 bibenzyl synthase-like [Dendrobium catenatum]
MPSLESIRKAPRANGFASILAIGRANPENFIEQSTYPDFFFRITNSEHLVDLKKKFQRICDKTAIRKRHF
VWNEEFITTNPCLHTFMDKSLDVRQEVAIREIPKLGAKAAAKAIQEWGQPKSRITHLIFCTTSGMDLPGA
DYQLTQILGLNPNVERVMLYQQGCFAGGTTLRLAKCLAESRKGARVLVVCAETTTVLFRGPSEEHQDDLV
TQALFADGASALIVGADPDEAAHERASFVIVSTSQVLLPDSAGAIGGHVSEGGLLATLHRDVPKIVSKNV
EKCLEEAFTPFGITDWNTIFWVPHPGGRAILDQVEERMGLKPEKLLVSRHVLAEYGNMSSVCVHFALDEM
RKRSAIEGKATTGEGLEWGVLFGFGPGLTVETVVLRSVHL

Dendrobium officinale bibenzyl synthase-like Protein（QCO76957.1）

>QCO76957.1 bibenzyl synthase-like protein [Dendrobium officinale]
MPSLESIRKAPRANGFASILAIGRANPENFIEQSTYPDFFFRITNSEHLVDLKKKFQRICDKTAIRKRHF
VWNEEFITTNPCLHTFMDKSLDVRQEVAIREIPKLGAKAAAKAIQEWGQPKSRITHLIFCTTSGMDLPGA
DYQLTQILGLNPNVERVMLYQQGCFAGGTTLRLAKCLAESRKGARVLVVCAETTTVLFRGPSEEHQEDLV
TQALFADGASALIVGADPDEAAHERASFVIVSTSQVLLPDSAGAIGGHVSEGGLLATLHRDVPKIVSKNV
EKCLEEAFTPFGITDWNSIFWVPHPGGRAILDLVEERVGLKPEKLLVSRHVLAEYGNMSSVCVHFALDEM
RKRSAIEGKATTGEGLEWGVVFGFGPGLTVETVVLRSVPL

Phalaenopsis equestris bibenzyl synthase-like（XP_020572025）

>XP_020572025.1 bibenzyl synthase-like [Phalaenopsis equestris]
MPSFPSVKKAPTAEGFASILAIGRANPENFIEQSAYPDFFFRVTNSEHLVDLKKKFQRICDKTAIRKRHF
VWNEEFLTANPCFSTFMDKSLNVRQEVAIREIPKLGAKAATKAIEDWGQPKSRITHLIFCTTSGMDLPGA
DYQLTQILGLNPNVERVMLYQQGCFAGGTTLRLAKCLAESRKGARVLVVCAETTTVLFRAPSEEHQDDLV
TQALFADGASAVIAVIVGADPDEAADERASFVIVSASQVLLPDSAGAIGGHVSEGGLLATLHRDVPQIVS
KNVGKCLEEAFTPFGISDWNSIFWVPHPGGRAILDQVEERVGLKPEKLSVSRHVLAEYGNMSSVCVHFAL
DEMRKRSAKEGKATTGEGLEWGVLFGFGPGLTVETVVLQSVPI

Cymbidium hybrid cultivar bibenzyl synthase（AIM58716）

>AIM58716.1 bibenzyl synthase [Cymbidium hybrid cultivar]
MPSLESVKKSNRADGFASILAIGRANPENFIEQSTYPDFFFRVTNSEHLVNLKKKFQRICDKTAIRKRHF
VWNEELLNANPCLGTFMDNSLNVRQEFAIREIPKLGAEAATKAIQEWGQPKSRITHLIFCTTSGMDLPGA
DYQLTQILGLNPNIERVMLYQQGCFAGGTTLRLAKCLAESRKGARVLVVCAETTAVLFRAPSEEHQDDLV
TQALFADGASALIVGADPDETAHERASFVIVSTSQVLLPDSAGAIGGHVSEGGLIATLHRDVPQIVSKNV
GKCLEEAFTPLGISDWNSIFWVPHPGGRAILDQVEERVGLKPEKLIVSRHVLAEYGNMSSVCVHFALDEM
RKRSKKEGKATTGEGLDWGVLFGFGPGLTVETVVLHSVPI

Dendrobium officinale bibenzyl synthase-like Protein（WOD46731.1）
>WOD46731.1 bibenzyl synthase-like protein [Dendrobium officinale]
MPSLESIKKAPRADGFASILAIGRANPENFIEQSAYPDLFFRITKSEHLVDLKNKFKRICDKTAIRKRHF
VWTEEFITANPCFSTFMEKSLNIRQEVAIREIPKLGAEAAAKAIQEWGQPKSRITHLIFCTRSGMGLPGP
DYQLTQILGLNPNVERVMIYQQGCFAGGTTLRLAKCLAESHKGARVLVVCAETSTVLFRAPSMEHQEDLV
TQALFADGASALIVGAHPDETADEHASFVIVSTSQVLLPESAGAIGGHVSEGGFLPMIHRDVPQIVSKNI
GKCLEEAFTPLGIMDWNSIFWVPHPGGRAILDQLDERVGLKPEKLFISRHVLKEYGNMSSASVHFALDEM
RKWSAKEGKGTTGEGLEWGVLFGFGPGVTVETVVLRSVPL
Bletilla striata chalcone synthase（AHH25569.1）
>AHH25569.1 chalcone synthase [Bletilla striata]
MPSLDSIKKAPRADGIASILAIGRANPDNIIEQSAYPDFYFRVTNSEHLVDLKKKFQRICEKTAIRKRHF
VWNEEFLTSNPSFSTFMDKSLYVRQEVAIREIPKLGAKAATKAIEDWGQPKSRISHLIFCTTSGMDLPGA
DYQLTQILGLNPNVERLMLYEQGCFAGGTTLRLAKCLAESRKGARVLVVCAETTTVLFRAPSEEHQDDLV
TQALFADGASALIVGADPDEAADERASFVIVSTSQVLLPDTAGAIGGHVSEGGLLATLHRDVPQIVTKNV
GKCLEEAFTPFGISDWNSIFWVPHPGGRAILDQVEERVGLKPEKLSVSRHVLAEYGNMSSVCVHFALDEM
RKRSANEGKPTTGEGLEWGVLFGFGPGLTVETVVLRSVPL
Phalaenopsis hybrid cultivar chalcone synthase（AAX54693.1）
>AAX54693.1 chalcone synthase [Phalaenopsis hybrid cultivar]
MPTIESIKKAPRAHGFASILAIGKANPENFIEQCHYPDFYFRVTSSEHLVDLKEKFQRMCDRTAIRKRHF
VWNEDLLTANPCLRTYMDKSLNIRQEVAIREIPKLGAEAATKAIQEWGQPKSSITHLIFCTTSGMDLPGA
DFQLTQILGLNPNVERVMLYQQGCFAGGTTLRLAKCLAESREGARVLVVCAETTTVVFRAPSEEHQDDLV
TQALFADGASAVIVGVDPNEAAHERASFIIVSASQVLLPDSAGAIGGHVSEGGLTATLHRDVPQIVSKNV
GKCLEEAFTPFGISDWNSIFWVPHAGGRAILDQVEERVGLKPEKLSVSRHVLAEYGNMSSVCVHFALDEM
RKKSAKEAKATTGEGLEWGVLFGFGPGLTVETVVLHSVPI
Polygonum cuspidatum PcPKS2 (ABY47640)
>ABY47640.1 type III polyketide synthase [Polygonum cuspidatum]
MAASIEEIRKEQTPATVLAIGTANPPNCLYQADFPDYYFRITKSDHLTHLKQKFKRICENSRIEKRYFQL
TEETIKENPNIGAYEAPSLNARHKIQVKGVAELGKEAALEAIKEWGQPKSKITHLIVCCLAGVDMPGTDY
QLTKLLDLHPTVKRFMFYHLGCYAGGTVLRLAKDIAENNKGARVLIVCSEMTAICFRGPSETNISSMIGT
SVLGDGAAAVIVGANPDLTVERPIFELVWTAQTIVPESDGAVEGHLLESGLSCHLSKTLPVLISNNIEAC
LSEAFTPLNISDWNSLFWITHPGGPAILDHVEAATGLNKEKLKATRQVLNDYGNMSSATVFFIMDKMRKR
SLENGRATTGEGLEWGVLFGIGPGVTVETVVLRSVPIIH
Rheum palmatum BAS (AAK82824)
>AAK82824.1 benzalacetone synthase [Rheum palmatum]
MATEEMKKLATVMAIGTANPPNCYYQADFPDFYFRVTNSDHLINLKQKFKRLCENSRIEKRYLHVTEEIL
KENPNIAAYEATSLNVRHKMQVKGVAELGKEAALKAIKEWGQPKSKITHLIVCCLAGVDMPGADYQLTKL
LDLDPSVKRFMFYHLGCYAGGTVLRLAKDIAENNKGARVLIVCSEMTTTCFRGPSETHLDSMIGQAILGD
GAAAVIVGADPDLTVERPIFELVSTAQTIVPESHGAIEGHLLESGLSFHLYKTVPTLISNNIKTCLSDAF
TPLNISDWNSLFWIAHPGGPAILDQVTAKVGLEKEKLKVTRQVLKDYGNMSSATVFFIMDEMRKKSLENG
QATTGEGLEWGVLFGFGPGITVETVVLRSVPVIS
Vitis vinifera STS(ABV82966)
>ABV82966.1 stilbene synthase [Vitis vinifera]
MASVEEIRNAQRAKGPATILAIGTATPDHCVYQSDYADYYFRVTKSEHMSELKKKFNRICDKSMIKKRYI
HLTEEMLEEHPNIGAYMAPSLNIRQEIITAEVPKLGKEAALKALKEWGQPKSKITHLVFCTASGVEMPGA

DYKLANLLGLETSVRRVMLYHQGCYAGGTVLRTAKDLAENNAGARVLVVCSEITVVTFRGPSEDALDSLV
GQALFGDGSAAVIVGSDPDVSIERPLFQLVSAAQTFIPNSAGAIAGNLREVGLTFHLWPNVPTLISENVE
KCLTQAFDPLGISDWNSLFWIAHPGGPAILDAVEAKLNLDKKKLEATRHVLSEYGNMSSACVLFILDEMR
KKSHKGEKATTGEGLDWGVLFGFGPGLTIETVVLHSIPMVTN

Rheum palmatum ALS (AAS87170)

>AAS87170.1 aloesone synthase [Rheum palmatum]
MADVLQEIRNSQKASGPATVLAIGTAHPPTCYPQADYPDFYFRVCKSEHMTKLKKKMQFICDRSGIRQRF
MFHTEENLGKNPGMCTFDGPSLNARQDMLIMEVPKLGAEAAEKAIKEWGQDKSRITHLIFCTTTSNDMPG
ADYQFATLFGLNPGVSRTMVYQQGCFAGGTVLRLVKDIAENNKGARVLVVCSEIVAFAFRGPHEDHIDSL
IGQLLFGDGAAALVVGTDIDESVERPIFQIMSATQATIPNSLHTMALHLTEAGLTFHLSKEVPKVVSDNM
EELMLEAFKPLGITDWNSIFWQVHPGGRAILDKIEEKLELTKDKMRDSRYILSEYGNLTSACVLFVMDEM
RKRSFREGKQTTGDGYEWGVAIGLGPGLTVETVVLRSVPIP

Gerbera hybrid 2-PS (CAA86219)

>CAA86219.2 2-pyrone synthase [Gerbera hybrid cultivar]
MGSYSSDDVEVIREAGRAQGLATILAIGTATPPNCVAQADYADYYFRVTKSEHMVDLKEKFKRICEKTAI
KKRYLALTEDYLQENPTMCEFMAPSLNARQDLVVTGVPMLGKEAAVKAIDEWGLPKSKITHLIFCTTAGV
DMPGADYQLVKLLGLSPSVKRYMLYQQGCAAGGTVLRLAKDLAENNKGSRVLIVCSEITAILFHGPNENH
LDSLVAQALFGDGAAALIVGSGPHLAVERPIFEIVSTDQTILPDTEKAMKLHLREGGLTFQLHRDVPLMV
AKNIENAAEKALSPLGITDWNSVFWMVHPGGRAILDQVERKLNLKEDKLRASRHVLSEYGNLISACVLFI
IDEVRKRSMAEGKSTTGEGLDCGVLFGFGPGMTVETVVLRSVRVTAAVANGN

Ruta graveolens ACS (CAC14058)

>CAC14058.1 acridone synthase [Ruta graveolens]
MESLKEMRKAQKSEGPAAILAIGTATPDNVYIQADYPDYYFKITKSEHMTELKDKFKTLCEKSMIRKRHM
CFSQEFLKANPEVCKHMGKSLNARQDIAVVETPRIGKEAAVKAIKEWGHPKSSITHLIFCTSAGVDMPGA
DYQLTRMLGLNPSVKRMMIYQQGCYAGGTVLRLAKDLAENNKGSRVLVVCSELTAPTFRGPSPDAVDSLV
GQALFADGAAALVVGADPDTSVERALYYIVSASQMLLPDSDGAIEGHIREEGLTVHLKKDVPALFSANID
TPLVEAFRPLGISDWNSIFWIAHPGGPAILDQIEVKLGLKEDKLRASKHVMSEYGNMSSSCVLFVLDEMR
NKSLQDGKSTTGEGLDWGVLFGFGPGLTVETVVLRSVPVEA

Sorbus aucuparia BIS (ABB89212)

>ABB89212.1 biphenyl synthase [Sorbus aucuparia]
MAPLVKNHGEPQHAKILAIGTANPPNVYYQKDYPDFLFRVTKNEHRTDLREKFDRICEKSRTRKRYLHLT
EEILKANPSIYTYGAPSLDVRQDMLNSEVPKLGQQAALKAIKEWGQPISKITHLIFCTASCVDMPGADFQ
LVKLLGLNPSVTRTMIYEAGCYAGATVLRLAKDFAENNEGARVLVVCAEITTVFFHGLTDTHLDILVGQA
LFADGASAVIVGANPEPKIERPLFEIVACRQTIIPNSEHGVVANIREMGFTYYLSGEVPKFVGGNVVDFL
TKTFEKVDGKNKDWNSLFFSVHPGGPAIVDQVEEQLGLKEGKLRATRHVLSEYGNMGAPSVHFILDDMRK
KSIEEGKSTTGEGLEWGVVIGIGPGLTVETAVLRSESIPC

Hypericum perforatum BPS (ABP49616)

>ABP49616.1 benzophenone synthase, partial [Hypericum perforatum]
MAPAMEYSTRNVQEEGKRRASVLAIGTTNPEHFILQEDYPDFYFKNTNSEHMTELKEKFKRICVKSHIRK
RHFYLTEDILKENQGIATYGAGSLDARQRILETEVPKLGQEAALKAIAEWGQPISKITHVVFATTSGFMM
PGADYAITRLLGLNRTVRRVMLYNQGCFAGGTALRVAKDLAENNANARVLVVCAENTAMTFHAPNESHLD
VIVGQAMFSDGAAALIVGANPDTSVGERAVFNILSASQTIVPGSDGAITAHFYEMGMSYFLKEDVIPLFR
DNIADVMKEAFSPLGVSDWNSLFYSIHPGGRGIIDGVAGNLGIKDENLVATRHVLGEYGNMGSACVMFIL
DELRRSSKLNGKPTTGDGKEFGCLIGLGPGLTVEAVVLQSVPILQ

Humulus lupulus VPS (BAA29039)
>BAA29039.1 valerophenone synthase [Humulus lupulus]
MASVTVEQIRKAQRAEGPATILAIGTAVPANCFNQADFPDYYFRVTKSEHMTDLKKKFQRMCEKSTIKKR
YLHLTEEHLKQNPHLCEYNAPSLNTRQDMLVVEVPKLGKEAAINAIKEWGQPKSKITHLIFCTGSSIDMP
GADYQCAKLLGLRPSVKRVMLYQLGCYAGGKVLRIAKDIAENNKGARVLIVCSEITACIFRGPSEKHLDC
LVGQSLFGDGASSVIVGADPDASVGERPIFELVSAAQTILPNSDGAIAGHVTEAGLTFHLLRDVPGLISQ
NIEKSLIEAFTPIGINDWNNIFWIAHPGGPAILDEIEAKLELKKEKMKASREMLSEYGNMSCASVFFIVD
EMRKQSSKEGKSTTGDGLEWGALFGFGPGLTVETVVLHSVPTNV
Hydrangea macrophylla STCS (AAN76182)
>AAN76182.1 stilbenecarboxylate synthase [Hydrangea macrophylla]
MATKSVAVEEMCKAQKAGGPATILAIGTAVPSNCYYQSEYPDFYFRVTKSDHLTDLKSKFKRMCDRSSIK
KRYMHLTEEILKENPNMCSFAAPSIDGRQDIVVKEIPKLAKEAASKAIKEWGQPESNITHLVFCTTSGVD
MPGCDYQLTRLLGLRPSIKRLMMYQQGCHAGGTGLRLAKDLAENNKGARVLVVCSEMTVINFRGPSEAHM
DSLVGQSLFGDGASAVIVGSDPDLSTEHPLYQIMSASQIIVADSEGVIDGHLRQEGLTFHLRKDVPSLVS
DNIENTLVEAFTPILMDSIDSIIDWNSIFWIAHPGGPAILNQVQAKVGLKEEKLRVSRHILSEYGNMSSA
CVFFIMDEMRKRSVEEGKGTTGEGLEWGVLFGFGPGFTVETIVLHSVPI
Marchantia polymorpha STCS2（AAW30009.1）
>AAW30009.1 stilbenecarboxylate synthase 1 [Marchantia polymorpha]
MANIVNAAAYKHRRAAGPATVLAIGKATPPTAYSQSEYPDFFFDITNTSHKTELKAKFARICKNSGINTR
YFHCTEDILKANPSMCTYLEPSLDVRQDIAIREVPRLAEKAAIEALAEWGQPRDQITHVVFATTSGVNMP
GADLTLTRLLGLNPNVKRTMLYQQGCFGGATVLRVAKDLAENNKGARVLTVVSELTCVTFRAPNEEHLDN
LVGSAIFGDGASVLVIGSDPIPEVEKPQFEIHWSGETILPESDGAIEGRLTEAGLIFHLLKDVPGLISRN
TLPIFNKAIEVAGSPSWNDLFWCVHPGGRAILDEVAKTLSLKPEKLEATRDILYNYGNMSGASVLFVLDQ
MRRRSAEKKSRTTGEGCEWGLVVGFGPGLTVEVSVLRAIATGH
Marchantia polymorpha STCS3（AAW30010.1）
>AAW30010.1 stilbenecarboxylate synthase 2 [Marchantia polymorpha]
MSRSRLIAQAVGPATVLAMGKAVPANVFEQATYPDFFFNITNSNDKPALKAKFQRICDKSGIKKRHFYLD
QKILESNPAMCTYMETSLNCRQEIAVAQVPKLAKEASMNAIKEWGRPKSEITHIVMATTSGVNMPGAELA
TAKLLGLRPNVRRVMMYQQGCFAGATVLRVAKDLAENNAGARVLAICSEVTAVTFRAPSETHIDGLVGSA
LFGDGAAAVIVGSDPRPGIERPIYEMHWAGEMVLPESDGAIDGHLTEAGLVFHLLKDVPGLITKNIGGFL
KDTKNLVGASSWNELFWAVHPGGPAILDQVEAKLELEKGKFQASRDILSDYGNMSSASVLFVLDRVRERS
LESNKSTFGEGSEWGFLIGFGPGLTVETLLLRALPLQQAERV
Hydrangea macrophylla CTAS (BAA32733)
>BAA32733.1 coumaroyl triacetic acid synthase [Hydrangea macrophylla]
MATKSVAVEEMCKAQKAGGPATILAIGTAVPSNCYYQSEYPDFYFRVTKSDHLTDLKSKFKRMCERSSIK
KRYMHLTEEILEENPNMCTFAAPSIDGRQDIVVKEIPKLAKEAASKAIKEWGQPKSNITHLVFCTTSGVD
MPGCDYQLTRLLGLRPSIKRLMMYQQGCHAGGTGLRLAKDLAENNKGARVLVVCSEMTVINFRGPSEAHM
DSLVGQSLFGDGASAVIVGSDPDLSTEHPLYQIMSASQIIVADSEGAIDGHLRQEGLTFHLRKDVPSLVS
DNIENTLVEAFTPILMDSIDSIIDWNSIFWIAHPGGPAILNQVQAKVGLKEEKLRVSRHILSEYGNMSSA
CVFFIMDEMRKRSMEEGKGTTGEGLEWGVLFGFGPGFTVETIVLHSVPI
Arachis hypogaea STS (BAA78617)
>BAA78617.1 stilbene synthase [Arachis hypogaea]
MVSVSGIRKVQRAEGPATVLAIGTANPPNCVDQSTYADYYFRVTNSEHMTDLKKKFQRICERTQIKNRHM
YLTEEILKENPNMCAYKAPSLDAREDMMIREVPRVGKEAATKAIKEWGQPMSKITHLIFCTTSGVALPGV

DYELIVLLGLDPSVKRYMMYHQGCFAGGTVLRLAKDLAENNKDARVLIVCSENTSVTFRGPSETDMDSLV
GQALFADGAAAIIIGSDPVPEVENPLFEIVSTDQQLVPNSHGAIGGLLREVGLTFYLNKSVPDIISQNIN
DALSKAFDPLGISDYNSIFWIAHPGGRAILDQVEEKVNLKPEKMKATRDVLSNYGNMSSACVFFIMDLMR
KKSLEAGLKTTGEGLDWGVLFGFGPGLTIETVVLRSMAI
Polygonum cuspidatum PcPKS1 (ABK92282)
>ABK92282.2 chalcone synthase [Polygonum cuspidatum]
MAPSVQEIRKAQRAEGPATVLAIGTATPPNCIYQADYPDYYFRVTNSEHMTDLKEKFRRMCDKSMIEKRY
MHLTEEILKENQNMCAYMASSLDSRQDMVVSEVPRLGKEAAQKAIKEWGQPKSKITHVIMCTTSGVDMPG
ADYQLTKLLGLRPSVKRFMMYQQGCFAGGTALRLAKDLAENTKGARVLVVCSEITAICFRGPTDTHLDSM
VGQALFGDGAGAVIIGADPDLSIERPIFELVWTAQTILPDSEGAIDGHLREVGLTFHLLKDVPGLISKNI
EKSLTEAFSPLNISDWNSLFWIAHPGGPAILDQVEAKLGLKEEKLKATRQVLNDYGNMSSACVLFIMDEM
RKKSLENGHATTGEGLDWGVLFGFGPGLAVETVVLHSVPVAHH
Cerbera hybrid cultivar CHS（CAA86218.1）
>CAA86218.1 chalcone synthase [Gerbera hybrid cultivar]
MASSVDMKAIRDAQRAEGPATILAIGTATPANCVYQADYPDYYFRITKSEHMVDLKEKFKRMCDKSMIRK
RYMHITEEYLKQNPNMCAYMAPSLDVRQDLVVVEVPKLGKEAAMKAIKEWGHPKSKITHLIFCTTSGVDM
PGADYQLTKLLGLRPSVKRFMMYQQGCFAGGTVLRLAKDLAENNKGARVLVVCSEITAVTFRGPNDTHLD
SLVGQALFGDGAAAVIVGSDPDLTTERPLFEMVSAAQTILPDSEGAIDGHLREVGLTFHLLKDVPGLISK
NIEKALTTAFSPLGINDWNSIFWIAHPGGPAILDQVELKLGLKEEKLRATRHVLSEYGNMSSACVLFIID
EMRKKSSENGAGTTGEGLEWGVLFGFGPGLTVETVVLHSVPTTVTVAV
Zea mays CHS（CAA42764.1）
>CAA42764.1 chalcone synthase [Zea mays]
MAGATVTVEEVRKAQRATGPATVLAIGTATPANCVYQADYPDYYFRITKSEHLTDLKEKFKRMCDKSMIR
KRYMHLTEEFLAENPSMCAYMAPSLDARQDVVVVEVPKLGKAAAQKAIKEWGQPKSRITHLVFCTTSGVD
MPGADYQLTKALGLRPSVNRLMMYQQGCFAGGTVLRVAKDLAENNRGARVLVVCSEITAVTFRGPSESHL
DSLVGQALFGDGAAAVVVGADPDDRVERPLFQLVSAAQTILPDSEGAIDGHLREVGLTFHLLKDVPGLIS
KNIGRALDDAFKPLGISDWNSIFWVAHPGGPAILDQVEAKVGLDKARMRATRHVLSEYGNMSSACVLFIL
DEMRKRSAEDGQATTGEGLDWGVLFGFGPGLTVETVVLHSVPITTGAATA
Vitis vinifera CHS（CAA53583.1）
>CAA53583.1 chalcone synthase [Vitis vinifera]
MVSVAEIRKAQRAEGPATVLAIGTATPANCVYQADYPDYYFRITNSEHMTELKEKFKRMCEKSMINKRYM
HLTEEILKENPNVCAYMAPSLDARQDMVVVEVPKLGKEAAAKAIKEWGQPKSKITHLVFCTTSGVDMPGA
DYQLTKLLGLKPSVKRLMMYQQGCFAGGTVLRLAKDLAENNAGSRVLVVCSEITAVTFRGPSDTHLDSLV
GQALFGDGAAAVIIGADPDTKIELPLFELVSAAQTILPDSEGAIDGHLREVGLTFHLLKDVPGLISKNIE
KSLVEAFTPIGISDWNSLFWIAHPGGPAILDQVELKLGLKEEKLRATRHVLSEYGNMSSACVLFILDEMR
KKSIEEGKGSTGEGLEWGVLFGFGPGLTVETVVLHSVSAPAAH
Scutellaria baicalensis CHS（BAA23373.1）
>BAA23373.1 chalcone synthase [Scutellaria baicalensis]
MVTVEEFHRATRAEGPATVLAIGTANPPNCVEQSTYADYYFRICKSEHLTDLKKKFDRMCEKSCIKKRYM
HLTEEFLKENDNFTAYEAPSLDARQDIVVVEIPKLGKEAAQKAIKEWGQPKSKITHVIFCTTSGVDMPGA
DYQITKLLGLRPSVKRFMMYQQGCFAGGTVLRMAKDLAENNAGARVLVVCSEITAITFRGPSDTHLDSLV
GQALFGDGAAAVIVGSDPIVGVERPLFQLVSAAQTILPDSEGAIDGHVREVGLTFHLLKDVPGLISKNIE
KSLKEAFAPLGISDWNSLFWIVHPGGPAILDQVEEKLGLKPEIMVPTRHVLSEYGNMSSACVLFVMDEMR
KASAKDGCTTTGEGKDWGVLFGFGPGLTVETVVLHSVPLN

Hydrangea macrophylla CHS（BAA32732.1）
>BAA32732.1 chalcone synthase [Hydrangea macrophylla]
MVTVEEVRKAQRAEGPATILAIGTATPPNYVDQSTYPDFYFRVTNSEHKKELKAKFQRMCDNSQIKKRYM
HLTEEILKENPNICAYMAPSLDARQDMVVVEIPKLGKEAATRAIKEWGQPKSKITHLVFCTTSGVDMPGA
DYQLTKLLGLRPSVKRLMMYQQGCFAGGTVLRLAKDLAENNKGARVLVVCSEITAVTFRGPSDTHLDSLV
GQALFGDGAAAVIIGSDPMPEVEKPLFEIVSAAQTILPDSDGAIDGHLREVGLTFHLLKDVPGLISKNIE
KSLVEAFRPLDISDWNSIFWIAHPGGPAILDQVEKKLALKPEKLRATRNVLSDYGNMSSACVLFIMDEMR
KNSAEEGLMTTGEGLEWGVLFGFGPGLTVETVVLHGVST
Medicago sativa CHS2（P30074）
>sp|P30074.1|CHS2_MEDSA RecName: Full=Chalcone synthase 2; AltName: Full=Naringenin-chalcone synthase 2
MVSVSEIRKAQRAEGPATILAIGTANPANCVEQSTYPDFYFKITNSEHKTELKEKFQRMCDKSMIKRRYM
YLTEEILKENPNVCEYMAPSLDARQDMVVVEVPRLGKEAAVKAIKEWGQPKSKITHLIVCTTSGVDMPGA
DYQLTKLLGLRPYVKRYMMYQQGCFAGGTVLRLAKDLAENNKGARVLVVCSEVTAVTFRGPSDTHLDSLV
GQALFGDGAAALIVGSDPVPEIEKPIFEMVWTAQTIAPDSEGAIDGHLREAGLTFHLLKDVPGIVSKNIT
KALVEAFEPLGISDYNSIFWIAHPGGPAILDQVEQKLALKPEKMNATREVLSEYGNMSSACVLFILDEMR
KKSTQNGLKTTGEGLEWGVLFGFGPGLTIETVVLRSVAI
